# Gastrointestinal microbiota and metabolites possibly contribute to distinct pathogenicity of SARS-CoV-2 proto or its variants in rhesus monkeys

**DOI:** 10.1080/19490976.2024.2334970

**Published:** 2024-04-02

**Authors:** Hongyu Chen, Junbin Wang, Kaiyun Ding, Jingwen Xu, Yun Yang, Cong Tang, Yanan Zhou, Wenhai Yu, Haixuan Wang, Qing Huang, Bai Li, Dexuan Kuang, Daoju Wu, Zhiwu Luo, Jiahong Gao, Yuan Zhao, Jiansheng Liu, Xiaozhong Peng, Shuaiyao Lu, Hongqi Liu

**Affiliations:** a Institute of Medical biology, Chinese Academy of Medical Sciences and Peking Union Medical School (IMBCAMS & PUMC), Kunming, Yunnan, China; b Institute of Laboratory Animal Sciences, IMBCAMS & PUMC, Beijing, China; c Institute of Basic Medical Sciences, IMBCAMS & PUMC, Beijing, China

**Keywords:** SARS-CoV-2, variant of concern, COVID-19, gastrointestinal infection, microbiota, integrative analysis

## Abstract

Gastrointestinal (GI) infection is evidenced with involvement in COVID-19 pathogenesis caused by SARS-CoV-2. However, the correlation between GI microbiota and the distinct pathogenicity of SARS-CoV-2 Proto and its emerging variants remains unclear. In this study, we aimed to determine if GI microbiota impacted COVID-19 pathogenesis and if the effect varied between SARS-CoV-2 Proto and its variants. We performed an integrative analysis of histopathology, microbiomics, and transcriptomics on the GI tract fragments from rhesus monkeys infected with SARS-CoV-2 proto or its variants. Based on the degree of pathological damage and microbiota profile in the GI tract, five of SARS-CoV-2 strains were classified into two distinct clusters, namely, the clusters of Alpha, Beta and Delta (ABD), and Proto and Omicron (PO). Notably, the abundance of potentially pathogenic microorganisms increased in ABD but not in the PO-infected rhesus monkeys. Specifically, the high abundance of *UCG-002*, *UCG-005*, and *Treponema* in ABD virus-infected animals positively correlated with interleukin, integrins, and antiviral genes. Overall, this study revealed that infection-induced alteration of GI microbiota and metabolites could increase the systemic burdens of inflammation or pathological injury in infected animals, especially in those infected with ABD viruses. Distinct GI microbiota and metabolite profiles may be responsible for the differential pathological phenotypes of PO and ABD virus-infected animals. These findings improve our understanding the roles of the GI microbiota in SARS-CoV-2 infection and provide important information for the precise prevention, control, and treatment of COVID-19.

## Introduction

Infection with severe acute respiratory syndrome coronavirus 2 (SARS-CoV-2) can cause Coronavirus Disease 2019 (COVID-19). Since it was first reported in late 2019, a global pandemic has rapidly developed, posing a serious threat to public health worldwide and the global economy.^1^ Distinguished from SARS-CoV and Middle East respiratory syndrome coronavirus (MERS-CoV), SARS-CoV-2 is highly transmissible and rapidly evolving. To date, more than 10 variants have been reported, particularly variants of concern (VOCs), such as B.1.1.7 (Alpha), B.1.351 (Beta), B.1.617.2 (Delta) and the recently reported B.1.1.529 (Omicron). These variants show distinct characteristics from the prototype strain (Proto), including transmissibility, immune escape, and even morbidity.^2,3^ The most common clinical symptoms observed in COVID-19 patients are fever, cough, shortness of breath, lung damage, and multi-organ failure to death occur in severe cases.^4^ Additionally, 30–40% of COVID-19 patients experience gastrointestinal (GI) symptoms such as abdominal pain, vomiting, nausea, and diarrhea. Furthermore, increasing evidences have shown that viral RNA can be detected in the stool of COVID-19 patients,^5^ which is supported by the high expression of ACE2-the major receptor for SARS-CoV-2 in the GI tract.^6^ Crucially, our previous study demonstrated that the GI tract is an alternative route of SARS-CoV-2 infection that induces apoptosis and inflammation in GI tissues.^7^

The complex symbiotic microbiota in the GI tract, is known as the “second genome” of hosts and is closely related to host nutrient absorption, growth, development, and also plays significant roles in host immunity against pathogens.^8^ Alterations in GI microbiota have been widely described in infectious diseases,^9^ including COVID-19.^10^ On the one hand, GI microbiota may mechanistically influence viral infection by interacting directly with viral particles such as bacterial stabilization of viral particles.^11^ On the other hand, the GI microbiota modulates the host immune system via its metabolites and indirectly affects the outcome of viral infection.^12^ For example, riboflavin derivatives, which are generally produced by *Bifidobacterium animalis*, *Bacteroides thetaiotaomicron*, and *Enterobacter cloacae*, activate mucosal-associated invariant T (MAIT) cells to rapidly promote an innate immune response to SARS-CoV-2 infection.^13,14^ Short-chain fatty acids (SCFAs) are one of the main metabolites produced by the GI microbiota that regulate immune cells to inhibit inflammation and enhance B cell activity.^15^ Impaired SCFA biosynthesis in the intestinal microbiota has been documented in COVID-19 patients, indicating the potential role of GI microbiota in SARS-CoV-2 infection.^16^ Recently, alterations in GI microbiota have been documented in hamster and non-human primate models of SARS-CoV-2 infection.^17,18^ Overall, GI microbiota is widely recognized to be involved in SARS-CoV-2 infection and associated with prognosis.^19^ Nonetheless, owing to limitations in sampling the GI tissues and their contents, and the complicated status of COVID-19 patients, how the GI microbiota contributes to COVID-19 remains unclear. Furthermore, determining whether the contribution of the GI microbiota to COVID-19 pathogenesis varies between SARS-CoV-2 and its variants is crucial.

Therefore, in this study, based on the non-human primate (NHP) model of SARS-CoV-2 infection we have established,^7^ we further analyzed the microbiota in GI contents and inflammation in tissues of each GI fragments from rhesus monkeys infected with SARS-CoV-2 proto or its recently reported four VOCs. We aimed to explore the impact that changes in the GI microenvironmental exert on SARS-CoV-2 pathogenesis infection. Overall, integrative results of multiomics analysis in this study revealed two distinct viral clusters based on their viral load, histopathology, immune response, and alteration of the microbiota. The findings of this study would facilitate our understanding the roles of the GI microbiota in SARS-CoV-2 infection and provide important information for the precise prevention, control, and treatment of COVID-19.

## Materials and methods

### SARS-CoV-2 prototype strain and variants

The five SARS-CoV-2 strains were used in this study, including the prototype strain (Proto), Alpha (B.1.1.7), Beta (B.1.351), Delta (B.1.617.2), and Omicron (B.1.1.529) that are kindly provided by institutes mentioned in acknowledgment under their Material Transfer Agreement. Viruses were cultured and amplified in Vero E6 cells. Viral culture for animal infection was ultrafiltered to replace the culture supernatant, with PBS using the Pellicon Ultrafiltration system (EMD Millipore) with a pore size of 300 nm. All procedures involving live viruses were performed in the ABSL-3 laboratory.

### Animal experiment procedures

**Figure 1.**
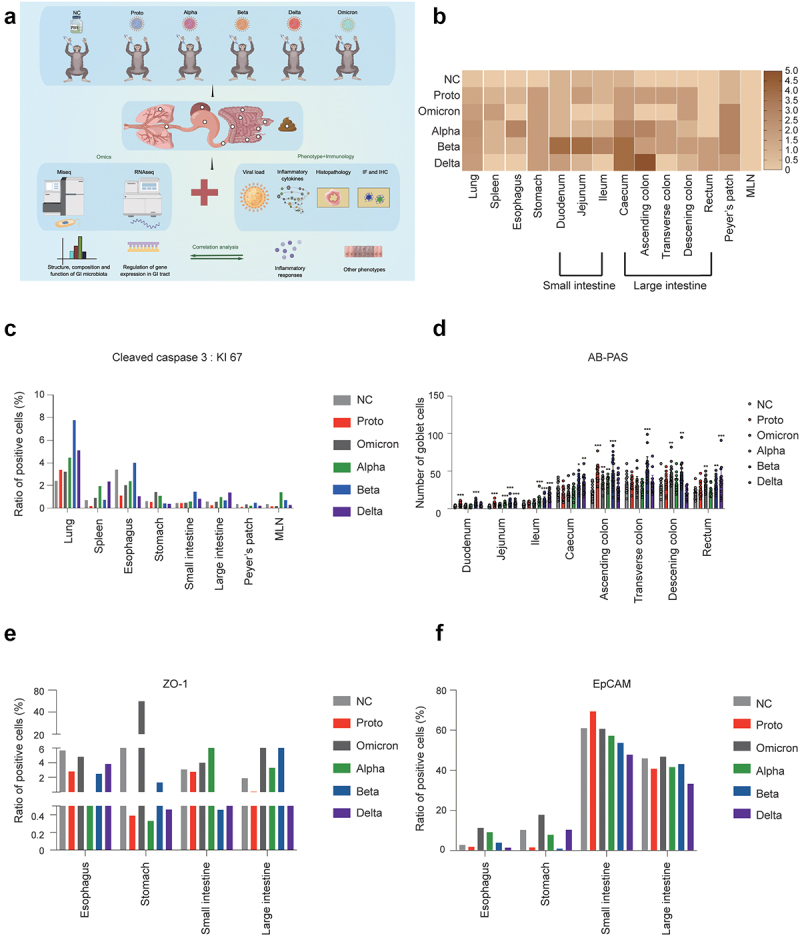
Histopathological analysis of pulmonary and GI tissues in rhesus monkeys challenged with SARS-CoV-2 or variants.

All animal procedures were approved (DWSP202102032) by the Institutional Animal Care and Use Committee of the Institute of Medical Biology at the Chinese Academy of Medical Science. Six rhesus monkeys were provided by the Kunming Primate Center of Chinese Academy of Medical Sciences (License #SCXK (Dian)-K2020–0005). The animals in this study are all health-screened, including exclusion of common pathogens, particularly SARS-CoV-2 and its antibody. After health screening, rhesus monkeys that met the criteria were randomly selected for each group. The animals were kept in cages separately (one animal/cage) to ensure as much as possible the safety of the experimenters and to prevent cross-infection among different viral strains. Animals were provided with free access to food and water and maintained under a 12-hour day/night cycle (light off at 8 PM). Animal information was summarized in Supplementary Table S1. Prior to every experimental operation, animals were anesthetized intramuscularly with ketamine (6 mg/kg body weight) according to the standard operation procedure of ABSL-3 laboratory. Viral inoculation at a dose of 10^7^ PFU was performed intranasally (0.5 ml, 10^7^ PFU/ml) and intratracheally (0.5 ml, 10^7^ PFU/ml), as previously described.^20^ At 5 days post infection (dpi), all animals were euthanized and dissected. The contents of the GI fragment were collected for viral load and microbiota analyses. Additionally, the GI tract, lung, and spleen tissues were harvested for histological and transcriptomic analyses (Figure 1(a)).

(a) Experimental design. Six rhesus monkeys were used in this study. Five monkeys were intranasally and intratracheally inoculated with SARS-CoV-2 Proto strain or indicated variants, respectively. One animal was treated with PBS as a negative control (NC). On the 5th day post treatment, all animals were euthanized and dissected for collection of GI contents, tissues of GI tracts, lung, and spleen, followed by evaluation of viral load, histological, transcriptomic or metagenomics analysis. Eventually, integrative analysis was conducted on the basis of phenotypes and omics. This figure was prepared via the online software figdraw (Copyright No. REG8221205045000212845). (b) Severity index of histopathology for the indicated tissues. The evaluation of severity index was described in Materials and Methods. (c) Apoptosis and proliferation of cells in indicated tissue samples were evaluated via immunohistochemical staining of the markers cleaved caspase-3 and KI-67. Ratio of staining density was calculated as described in Materials and Methods. Representative images were shown in Fig. S3A, B. (d) Mucosal barrier integrity of GI tract was evaluated by AB-PAS staining as described in Materials and Methods. Compared with the NC, significant differences in goblet cell count in GI tissues of variants of infected animals was indicated by *One-way ANOVA*. *(*p < .05, ** *p* < .01, *** *p* < .005, **** *p* < .001). Representative images were attached in Fig. S3C. (e and f) The epithelial integrity of the GI tract was determined via immunofluorescence staining of Zonula occludens protein (ZO-1) and epithelial cell adhesion molecule (EpCAM) as described in Materials and Methods. Representative images were shown in Fig. S3D.

### Determination of viral load in GI tissues

The Direct-zol RNA Miniprep Extraction Kit (Zymo Research, Catalog no. R2052) was used to extract the viral RNA from the collected tissues according to the manufacturer’s instructions. Genomic RNA of SARS-CoV-2 was quantified using qRT-PCR with previously described primers and probes.^7^

### Histopathologic analysis

The collected tissue samples were fixed in 10% neutral buffered formalin for at least 7 days. Three-micrometer sections were prepared for hematoxylin and eosin (H&E) staining and histopathological analysis. After H&E staining, the slides were scanned using a 3D HISTECH (Hungary) dataset and viewed via the CaseViewer software. An experienced pathologist examined the whole section for histological changes at different magnifications and scored each section on the basis of the histological lesions (0, absent; 1, slight; 2, mild; 3, moderate; 4, severe) and inflammatory infiltration (0, absent; 1, slight; 2, mild; 3, moderate; 4, severe). The sum of both scores produced the severity index for each slide.

### Immunohistochemical analysis

The tissue samples were embedded in paraffin and cut into 3 μm sections, and immunohistochemical staining was performed to detect specific markers using the following antibodies (obtained from Servicebio): anti-cleaved caspase-3 (Catalog no. GB13436), anti-Ki67 (Catalog no. GB111499), and anti-CD68 (Catalog no. GB113150). The stained slides were scanned using the 3D HISTECH system. Positively stained cells on each slide were identified using the artificial intelligence image analysis software Aipathwell (Service Bio). The staining index (%) was calculated as the percentage of positive cells out of the total number of cells. The log_2_ fold change (infected monkeys vs NC) in the staining index was plotted as a heatmap using Graph Pad Prism 8.0.

### Immunofluorescence analysis

The tissue samples were embedded in paraffin, cut into 3 μm section and incubated with the following primary antibodies: anti-ZO-1 (ThermoFisher, Catalog no. 33–9100; 1:100), anti-EpCAM (Abcam, Catalog no. ab282457; 1:100), anti-CD4 (Abcam, Catalog no. ab133616; 1:5000) and anti-CD8 (Abcam, Catalog no. ab237709; 1:5000). Then, the corresponding fluorescent-conjugated secondary antibodies were added to the sections, which were counterstained with DAPI and scanned by the 3D HISTECH system (3DHISTECH). The positively stained cells on each slide were identified via the artificial intelligence image analysis software Aipathwell (Service Bio). The staining index (%) was calculated for each slide as the percentage of positive cells divided by the total number of cells. The log2 fold change (infected monkeys vs. NC) in the staining index was plotted as a heatmap using GraphPad Prism 8.0.

### Alcian blue and periodic acid – Schiff staining

The GI tissue fragments were embedded in paraffin, cut into 3 μm sections, and stained with Alcian blue and Periodic Acid-Schiff (AB-PAS) by a staining kit (Catalog no. G1049, Solarbio) according to the manufacturer’s instructions. The stained slides were scanned by the 3D HISTECH system, and the goblet cells in each slide were counted as described previously.^7^ Briefly, the slides were equally divided into four parts, and five crypts were randomly selected to count the goblet cells. Only goblet cells at a distance 150 μm above distance from the surface epithelium of the crypts (longitudinally cut) were counted.

### Multiplex assay of inflammatory cytokines

A MILLIPLEX MAP Non-Human Primate Cytokine Magnetic Bead Panel-Immunology Multiplex Assay (Millipore) was performed on the GI fragments and spleen tissues in a Bio-Plex machine (Bio-Rad) according to the manufacturer’s instructions. The cytokines in this panel were grouped as immunoregulatory cytokines, including interleukin (IL)-1α, IL-2, IL-4, IL-5, IL- 10, IL-15, interferon gamma (IFN-γ), granulocyte colony-stimulating factor (G-CSF), granulocyte-macrophage colony-stimulating factor (GM-CSF), and inflammatory cytokines such as IL-1β, IL-6, IL-8, IL-12/23(P40), IL-17A/CTLA8, IL-18, MCP-1/CCL2, MIP-1α/CCL3, MIP-1β/CCL4, tumor necrosis factor alpha (TNF-α), transforming growth factor alpha (TGF-α), and vascular endothelial growth factor (VEGF).

### 16S rRNA sequencing analysis of microbiota in contents of GI fragments

The contents of the GI fragments and feces from each animal were collected directly into a DNA/RNA Shield^TM^ Lysis Tube (ZymoBIOMICSTM, Catalog no. R1103–50). The total DNA of the bacterial genome was extracted via a DNA Miniprep Kit (ZymoBIOMICSTM, Catalog no. D4300) according to the manufacturer’s instructions, followed by confirmation of extracted DNA via 1.0% agarose gel electrophoresis and determination of DNA concentration on a NanoDrop ND-2000 spectrophotometer (Thermo Scientific Inc., USA). The bacterial 16S rRNA gene (V3-V4 hypervariable regions) was amplified by PCR with primer pairs 338F (5’-ACTCCTACGGGAGGCAGCAG-3’) and 806 R (5’-GGACTACHVGGGTWTCTAAT-3’) on an ABI GeneAmp® 9700 PCR thermocycler (ABI, CA, USA). The PCR reaction mixture was prepared as follows: 4 μL 5 × Fast Pfu buffer, 2 μL 2.5 mM dNTPs, 0.8 μL of each primer (final concentration 5 μM), 0.4 μL Fast Pfu polymerase, 10 ng of template DNA, and ddH_2_O to a final volume of 20 µL. PCR amplification was conducted under the following conditions: initial denaturation at 95°C for 3 min, followed by 30 cycles of denaturation at 95°C for 30s, annealing at 45°C for 30s and extension at 72°C for 45s, and single extension at 72°C for 10 min, kept at 10°C until halted by user.

The PCR products were purified by an AxyPrep DNA Gel Extraction Kit (Axygen Biosciences, Catalog No. AP-GX-250 G) and quantified in a Quantus fluorometer (Promega, USA). Libraries were constructed via the NEXTFLEX Rapid DNA-Seq Kit (Bio Scientific, Catalog no. NOVA-5144) and sequenced on an Illumina PE300 instrument with assistance from Majorbio Bio-Pharm Technology Co., Ltd. (Shanghai, China). The sequencing data were submitted to the Sequence Read Archive of the National Center of Biotechnology Information (accession number: PRJNA894807). The raw sequencing reads of the 16S rRNA gene were demultiplexed and quality-filtered using fastq (version 0.20.0) and merged using FLASH (version 1.2.7) as follows: i) a 50 bp sliding window to the truncated site with an average quality score of < 20; ii) overlapping sequences longer than 10 bp were assembled according to their overlapping sequence (maximum mismatch ratio: 0.2). After quality control was performed, the clean reads with 97% similarity were clustered into operational taxonomic units (OTUs) and chimeric sequences were culled using UPARSE V11.0.667_i86osx32 (http://drive5.com/usearch/download.html). The number of sequences in each sample was normalized to the minimum number of sequences. The OTUs were taxonomically classified against the Silva 138 database (confidence threshold: 0.7) using the RDP Classifier (version 2.2). The rarefaction curve and alpha-index (Sobs and Shannon indices) were calculated using Mothur (version 1.40.5) and plotted using the R package. Principal coordinate analysis (PCoA) was performed on basis of the distance algorithms “abund_jaccard” using the Vegan v2.5–3 package, and the statistical significance of the clusters was determined by PERMANOVA (permutational analysis of variance). The correlation between multiplex factors (cytokines and DEGs) and microorganisms was determined on basis of distance-based redundancy analysis (db-RDA) via Vegan v2.5–3 and pheatmap packages, respectively. The metagenomic function was predicted by phylogenetic investigation of communities via reconstruction of unobserved states (PICRUSt2). Matrix correlations were analyzed using the R pheatmap package.

### Transcriptomic analysis via illumina sequencing

Total RNA in the tissues (50–60 mg) was extracted by the RNeasy Universal Kits (QIAGEN, Catalog no. 73404) according to the manufacturer’s instructions. RNA concentration was measured in a NanoDrop 2000 (Thermo Scientific) and RNA quality was evaluated by an Agilent 2100 Nano. High-quality RNA (OD260/280 = 1.8–2.2, OD260/230 ≥ 2.0, RIN ≥ 6.5, and 28S:18S ≥ 1.0) was used to construct a library for sequencing. In total 1 μg of RNA was used to construct the RNA-seq library via the Illumina TruSeq^TM^ RNA sample preparation Kit (Illumina, Catalog no. 20040532) according to the manufacturer’s instructions. Briefly, mRNA isolated by oligo(dT) was randomly fragmented into approximately 300bp segments using a fragmentation buffer. cDNA was synthesized using a SuperScript double-stranded cDNA synthesis kit (Invitrogen, Catalog no. 11917020) with random hexamer primers (Illumina). Next, the ends of the double-stranded cDNA were aligned by End Repair Mix. End repair was performed according to Illumina’s library construction protocol that includes phosphorylation and addition of the ‘A’ base. The target fragments (300 bp cDNA) were further screened in 2% Low Range Ultra Agarose and amplified by PCR. After quantification on the QuantiFluor® dsDNA System, the paired-end RNA-seq libraries were sequenced on an Illumina NovaSeq6000.

After sequencing, assessment and quality control of the raw data were conducted by SeqPrep (https://github.com/jstjohn/SeqPrep) and Sickle (https://github.com/najoshi/sickle) to remove adapters and low-quality reads (such as reads with less than 20bp in the 3’-end or containing more than 10% ambiguous bases “N.”). The clean reads were assembled and mapped (reference genomes Macaca mulatta Mmul_10 [http://www.ncbi.nlm.nih.gov/data-hub/taxonomy/9544/]) via StringTie V2.2.0 (https://ccb.jhu.edu/software/stringtie/index.shtml?), and HISAT-3N (https://daehwankimlab.github). io/hisat2/), respectively.

Gene expression was calculated in units of fragments per kilobase of exon per million mapped reads (FPKM) by RSEM v1.3.3. The analysis of differentially expressed genes (DEGs) was conducted using the R statistical package software EdgeR (Empirical analysis of Digital Gene Expression in R). Significant differences under the threshold: log_2_| fold change |≥2; false discovery rate (FDR) ≤ 0.05). The functions and pathways of genes were annotated by the Gene Ontology (GO, http://www.geneontology.org/) and Kyoto Encyclopedia of Genes and Genomes (KEGG: http://www.genome.jp/kegg/) databases, GOatools (https://github.com/tanghaibao/GOatools), and KOBAS 3.0(http://kobas.cbi.pku.edu.cn/), respectively.

Based on the DEGs profile, the genes associated with traits (GI segments of NC and infected monkeys) were further identified by weighted gene co-expression network analysis (WGCNA) via the R package as follows: genes with low expression levels (relative expression levels < 1) and coefficient of variation (<0.1) were filtered. Subsequently, the optimal power values of the genes were calculated as the soft power (beta) for the adjacency matrix. The genes were then further clustered and divided into modules (networkType: signed; soft power:6; minModuleSize:50; minKMEtoStay:0.3; mergeCutHeight:0.20). The genes that were not clustered into any module are indicated in “gray” color. Correlation analysis was performed by Spearman’s correlation coefficients for discontinuous data types. Finally, the genes with a high correlation module (R^2^ > |0.5|, *p* < .05) were further filtered to screen for candidate hub genes.

Gene set enrichment analysis (GSEA) was performed to identify the leading edge DEGs. In this study, the immune and infectious disease pathways in KEGG were used to evaluate enriched pathways via the GSEA software (v4.0.3, http://www.broadinstitute.org/gsea) as a gene set. Subsequently, mRNA expression profiles were used as inputs. The following parameters were used: number of permutations = 1000; permutation type= gene_set (biological replicates less than seven); metric for ranking genes: Signal2Noise. A gene set was considered significantly enriched if its normalized enrichment score (NES) was above 1 (*p* < .05).

### DEG validation using quantitative real-time PCR (qRT-PCR)

DEGs were validated using qRT-PCR, including randomly selected six genes (*LCN2*, *MUC2*, *MNP2*, *DEFA4*, *MAMU-AG* and *GZMB*). The RNA samples used for qRT-PCR were from RNA originally used for RNA sequencing. The reverse transcription conditions were as follows: initial denaturation at 95°C 5 min, followed by 35 cycles of denaturation at 95°C 30s, then extension at 72°C 1 min. The primer sequences used in this study were as follows: LCN2-F GGGAAGTGGTATGTGGTAG, LCN2-R ATGGAGGTGACGTTGTAGC; MUC2-F CTGGGATTTGAAGCGAAGA, MUC2-R TTGGAGGAATAAACTGGAGAA; MNP2-F AAGTCACTCCAGGAAACAGC, MNP2-R ACTCACGCCTGAAACAACG; DEFA4-F CTGCCATTCTCCTCTTCAC, DEFA4-R GACAGCAGAGTTTGTGGGT; MAMU-AG-F AGAACGGGAAGGAGACGC, MAMU-AG-R CCAGAAGGCACCACCACA; GZMB-F AGAGGACTTCGTGCTGACA, GZMB-R GATTATAGGCTGGGTGGG. GAPDH was used as the reference gene for normalization (GAPDH-F GGACCTGACCTGCCGTCTA, GAPDH-R GAGTGGGTGTCGCTGTTGA). The amplification conditions for PCR were as follows: initial denaturation at 95°C for 5 min, followed by 40 cycles of denaturation (95°C for 5s), annealing (55°C for 30s) and extension (72°C for 40s). The melting curves were generated from 60°C–95°C. All reactions were performed with three technical replicates to calculate the fold change by 2^−ΔΔCT^.

### Integrative analysis of GI microbiome and transcriptome

To explore the correlations between alterations in the GI microbiota and transcriptomic changes in the GI tissues of SARS-CoV-2 infection, matrix correlation analysis was conducted using the R psych package. The top 20 most abundant microbial genera were selected for correlation analysis against the infection- and immunity-associated DEGs (such as for interleukin, integrin, and addressin, antiviral, antibacterial, and IBD-associated genes). The correlations were calculated via the Spearman’s correlation coefficient.

### Statistical analysis

The R stats were tested for differences among the groups. The Kruskal–Wallis H test was used for the multi-group tests. Charts, including partial lines, heat maps, and scatter diagrams, were prepared via Graph Pad Prism 8.0. The details of all the statistical analysis methods used in this study are shown in the figure legends.

## Results

### GI dysfunction associated the histopathologic changes and inflammation induced by infection with SARS-CoV-2 proto or its variants

To determine the effects of SARS-CoV-2 infection on the respiratory and GI systems, tissues from the lungs and fragments of the GI tracts were harvested on day 5 post infection with the SARS-CoV-2 prototype or its VOCs (Figure (1a)). In the respiratory system, all the tested SARS-CoV-2 strains caused histopathological damage, mainly including epithelial cells detachment, alveolar dilatation and fusion, and infiltration of inflammatory cells around the blood vessels. In the GI system, viral infection resulted in the infiltration of inflammatory cells and necrolysis, particularly in the large intestines and gut-associated lymphoid tissues (Peyer’s patches) of Delta-, Beta-, and Alpha-infected animals (Figure S1). Among these viral strains, Beta-infection resulted in the highest histopathology severity index, followed by Delta, Alpha, Proto, and Omicron variants (Figure (1b)). The high ratio of KI-67 and cleaved caspase 3, assayed by immunohistochemical staining, suggested that apoptosis was significantly induced in pulmonary and GI tissues of SARS-CoV-2 infection, especially Alpha-, Beta-, and Delta-infected monkeys (Figure (1c)) and Fig. S3A, B). At transcriptional level, the upregulation of *CASP3* and downregulation of *KI67* (except for Proto strain) were observed in infected animals (Supplementary Table S2). Viral loads were consistently higher in the GI tissues and contents from the Delta-, Alpha-, and Beta-infected monkeys than the Proto- and Omicron-infected monkeys (Fig. S2A). Furthermore, transcriptomic analysis revealed that the major receptor *ACE2* was primarily and abundantly expressed in small intestine as well as Peyer’s patches, although ACE2 mRNA was also detected in other fragments of the GI tract (Fig. S2B), which is consistent with results from human samples.^21^ Moreover, *TMPRSS2* and *ADAM7* were widely expressed in the GI tract (Fig. S2C and D). Therefore, infections of SARS-CoV-2 Proto or its variants cause various degrees of histopathological changes in fragments of GI tract in rhesus monkeys.

Function of the GI tract was then evaluated by AB-PAS staining of mucin, immunofluorescence staining of Zonula occludens protein (ZO-1) and epithelial cell adhesion molecule (EpCAM) (Figure 1(d–f) and Fig. S3C, D). AB-PAS staining showed that the mucin levels gradually increased from the duodenum to the rectum in each GI tract, which is consistent with data from the human GI tract.^22^ However, comparison among the tested viral strains showed that high mucin levels were observed in GI fragments of Alpha-, Beta-, and Delta-infected animals (Figure 1(d)). The distribution of mucin genes in the GI tract shown at the transcriptional level was similar to results in AB-PAS staining (Fig. S3E). As expected, immune-related tissues (Peyer’s patches and MLN) showed expression of most mucin genes. Furthermore, *MUC1* and *MUC20* were significantly and negatively correlated with immune-related tissues from Alpha-, Beta-, Delta- or Omicron-infected animals. Following infection with SARS-CoV-2, multiple mucin genes showed significantly positive correlations in the digestive tissues, particularly *MUC1*, *MUC12* and *MUC13*. ZO-1 and EpCAM contribute to the integrity and permeability of the GI tract.^23^ We observed that infection with the Delta or Beta variant resulted in decreased expression of ZO-1 and EpCAM in both the small intestine and large intestine, with no difference between Omicron and NC (Figure 1(e, f)). Consistently, genes associated with GI function (*MUC2*, *TJP1*, *TJP2*, and *EPCAM*) were upregulated in the GI tissues of the infected monkeys (Supplementary Table S2). Therefore, these results demonstrate that SARS-CoV-2 Proto or its variant infection-induced mucin and barrier dysfunctions of the GI tract in rhesus monkeys.

**Figure 2.**
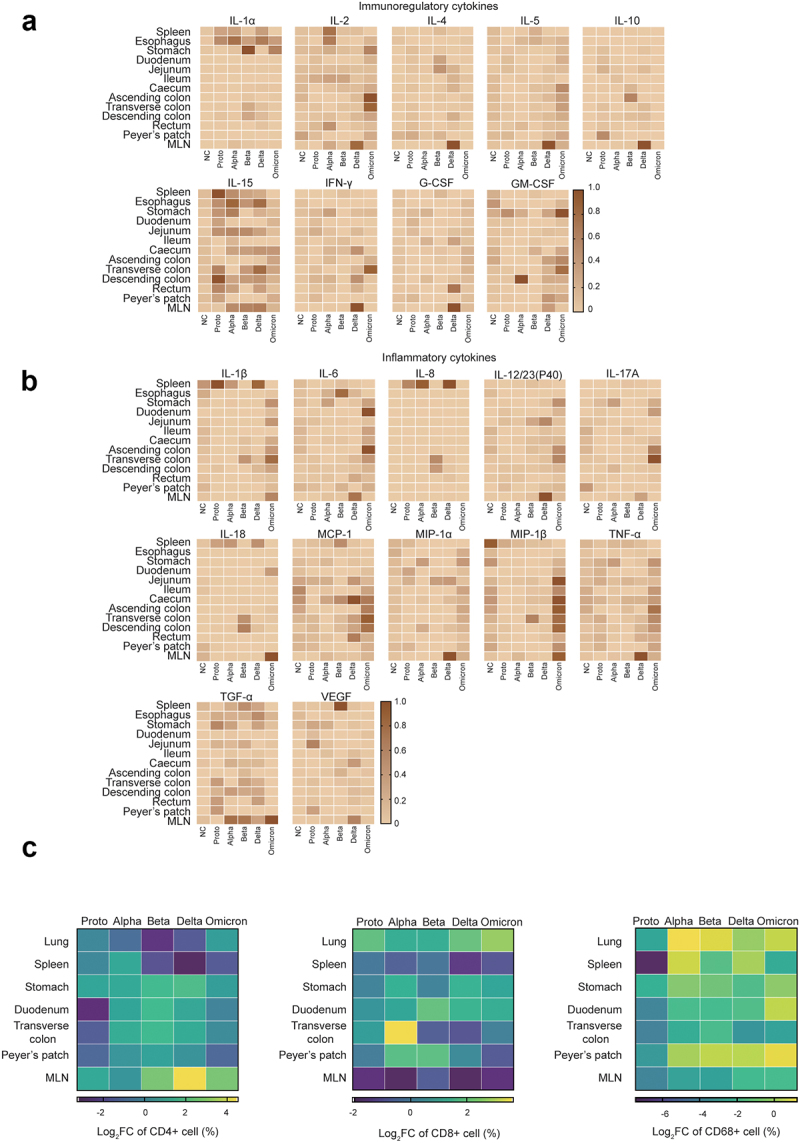
Inflammatory responses in GI tracts post SARS-CoV-2 proto strain or variants.

Next, we asked whether GI dysfunction is associated with the inflammatory environment induced by infections of SARS-CoV-2 Proto and its variants. A multiplex cytokine assay was first performed and showed that SARS-CoV-2 infections induced some immunoregulatory (IL-1α, IL-2, IL-15, IFN-γ, and GM-CSF) and inflammatory cytokines (IL-1β, IL-6, IL-23, MCP-1, MIP-1β, TNF-α, TGF-α and VEGF) in the GI tract fragments and spleen at 5 dpi (Figure 2(a,b). IL-15 was widely expressed in various tissues. In contrast, cytokines IL-1α, IL-2, IL-1β, IL-8, IL-18, and TGF-α were preferentially induced in the spleen and mesenteric lymph nodes (MLN). Nearly all the analyzed cytokines were highly induced in the MLN from Delta-infected animals. Unexpectedly, several cytokines were massively induced in GI tract and other tissues by Omicron infected, including IL-1β, IL-6, IL-12/23(P40), IL-17A, MCP-1, MIP-1β, TNF-α, and some immunoregulatory cytokines (IL-2, IL-5, GM-CSF, and IFN-γ). To determine the association of the cytokine milieu with the types of immune cells, we then analyzed levels of CD4+ and CD8+ cells in the tissue samples (Figure 2(c) and Fig. S4A). The ABD-infected monkeys, in particular, showed an increased number of CD4+ cells in the stomach, duodenum, transverse colon and Peyer’s patches. Similarly, CD8+ cell number increased in the lungs, stomach, duodenum, and Peyer’s patches but decreased in MLN. In comparison, ABD-infected monkeys showed higher proportions of CD4+ and CD8+ cells in multiple GI tissues than the PO-infected monkeys, suggesting an association between cytokine milieu and these immune cells. Furthermore, the infected rhesus monkeys exhibited an increase in CD68 expression in the lungs, spleen, and Peyer’s patches post SARS-CoV-2 infection, although its expression was relatively low in GI fragment tissues. In these tissues, CD68 expression was higher in the ABD- than in the PO- infected animals (Figure 2(c) and Fig. S4B). Taken together, infections with the SARS-CoV-2 strains induce distinct inflammatory responses in the GI tissues, which probably explains various degrees of histopathological changes and GI dysfunction observed in SARS-CoV-2 Proto or its variants in infected animals.

On 5 dpi, animals were euthanized and dissected. Tissues were collected for analysis of cytokines via the commercial kit as described in Materials and Methods. According to reported functions of cytokines, cytokines were divided into two panels, immunoregulatory (a) and inflammatory (b) cytokines. The highest cytokine level is set as 1 for normalization. The relative levels of cytokines were expressed in terms of color density in heatmaps. (c) Expressions of CD4 (left), CD8 (middle) and CD68 (right) in slides of indicated tissue samples were shown via heatmap of log_2_ fold change (infected monkeys vs NC) of staining index that was calculated as described in Methods. This figure was created via the GraphPad Software. Representative images were shown in Fig. S4.

### Distinct profiles of GI microbiota and metabolites correlated with GI immune microenvironment induced by infections of SARS-CoV-2 proto or its variants

To explore the involvement of the GI microbiota in SARS-CoV-2 pathogenesis, we characterized the microbiome in the contents of GI fragments from monkeys via high-throughput sequencing of the microbial 16S ribosomal RNA gene (V4-V5). In total, 1,308,997,036 bases were sequenced, out of which 3,116,212 quality-filtered effective sequences were acquired from 59 samples. We identified 1,795 operational taxonomic units (OTUs) that was annotated to 22 bacterial phyla, 47 classes, 111 orders, 201 families, 497 genera, and 916 species. Microbiota alpha diversity and majority of microbial genera in GI fragments from uninfected rhesus monkeys (Fig. S5) were similar to the GI microbiota profile in humans,^24^ suggesting that rhesus monkeys are a suitable model for investigation of human GI microbiota. Furthermore, among the top 20 genera in the GI tract, the microbial abundance in feces was similar to that in the large intestine but extremely different from that in the small intestine and stomach. Particularly, *Streptococcus* was abundant in the small intestine, but few in the large intestine and feces, whereas *Alloprevotella* and *Faecalibacterium* were abundant in the large intestine, but rarely detected in the feces and small intestine (Fig. S5C, D, E). These results demonstrated that contents from different fragments in the GI tract instead of feces only need to be subjected to microbiome analysis to comprehensively clarify the GI microbial profile.

**Figure 3.**
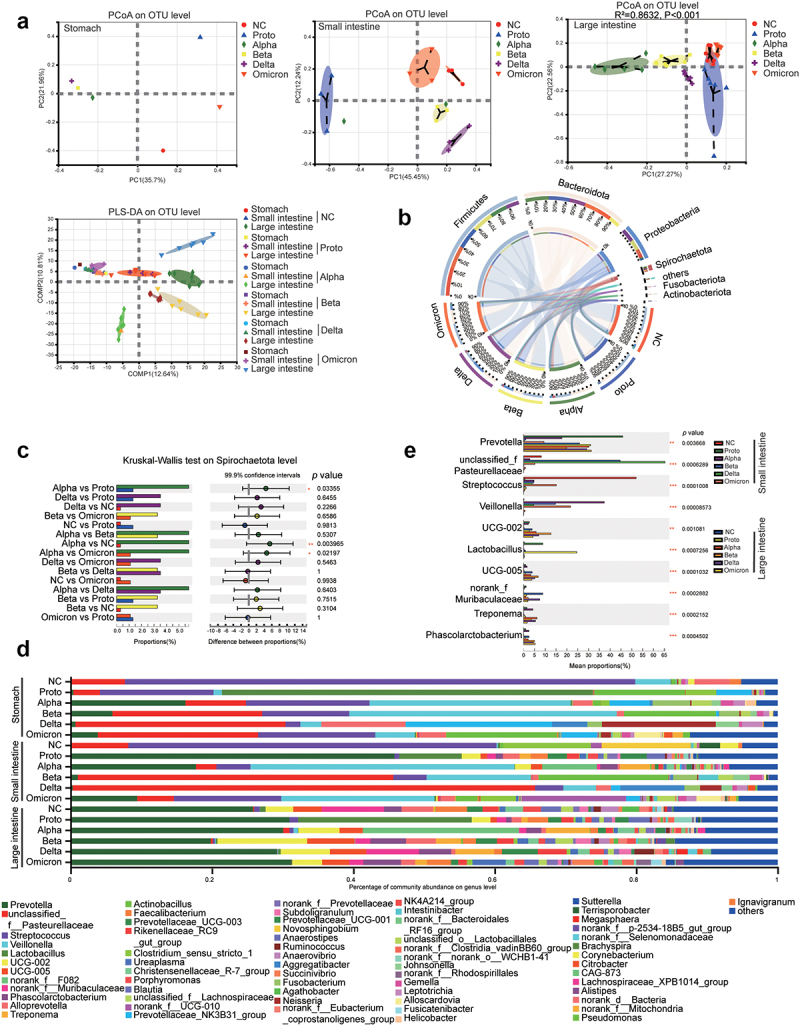
Microbial beta-diversity, composition and dominant microorganisms in GI contents of SARS-CoV-2 or variants infected animals.

We then observed that the alpha diversity of the GI microbiota was altered post infection and differed among SARS-CoV-2 prototypes or its four variants, particularly in large intestines of infected monkeys (Fig. S6A). Delta strain infection induced significant reductions in alpha diversity (Sobs and Shannon index) in both the small and large intestines (*p* < .05), which was the lowest among all the VOCs (Fig. S6A). Furthermore, Alpha or Delta infection increased the proportion of potentially pathogenic microorganisms in the GI tract of infected monkeys (Fig. S6B). Beta diversity by Principal coordinate analysis (PCoA) and partial least squares discriminant analysis (PLS-DA) showed that after infection, the microbiota in both the small intestines and large intestines of Alpha-, Beta- and Delta-infected monkeys formed a cluster that was distinct from those of the NC, Proto, or Omicron-infected animals (significantly in large intestine R^2^ >0.5; *p* < .05) (Figure 3(a)). At the phylum level, the top three microbes in the NC and infected monkeys were Firmicutes, Bacteroidetes, and Proteobacteria (Figure 3(b)), which is consistent with results previously reported in humans and monkeys.^25^ Interestingly, a high abundance of Spirochaetota was observed in SARS-CoV-2 infected monkeys, particularly in the Alpha-, Beta-, or Delta-infected animals (Figure 3(b, c)). At the genus level, *Prevotella* was the most abundant in the large intestines of both NC and SARS-CoV-2 infected monkeys (Figure 3(d)), confirming the accuracy of the sequencing and annotation in this study. Notably, among the top 15 genera, the abundance of *Veillonella* (in the small intestine) and *Treponema* (in the large intestine) significantly increased post SARS-CoV-2 infection (*p* < .05), whereas that of *Lactobacillus* significantly decreased (Figure 3(e)). These results suggested that distinct microbiota profile is caused by infections of SARS-CoV-2 Proto or its variants.

On 5 dpi, animals were euthanized and dissected. GI contents from each fragment of the GI tract were collected for analysis of microbiota via 16S rRNA sequencing as detailed in Materials and Methods. (a) Principal coordinate analysis (PCoA) and partial least squares discriminant analysis (PLS-DA). The significant difference among clusters was analyzed via PERMANOVA (Permutational analysis of variance). R^2^ was used to interpret the degree of difference among groups. *p* < .05 means significant difference among groups. (b) Composition of GI microbes at the phylum level was plotted by Circos-0.67-7 (http://circus.ca/). The left half-circle ribbons outside and inside represent sample compositions and proportion of phyla, respectively. The right half-circle ribbons outside and inside represent distribution proportion of each phylum in group and relative abundance of the phylum in each group. (c) Difference of relative abundance of the phylum Spirochaetota among groups (*Kruskal-Wallis H test*; *p < .05; **p < .01; ***p < .005; ****<0.001). (d) Composition of GI microbes at the genus level. (e) The top 10 bacterial genera with significant difference in relative abundance (mean) among viral infection groups. (*Kruskal–Wallis H test*; *p < .05; **p < .01; ***p < .005; ****p < .001).

**Figure 4.**
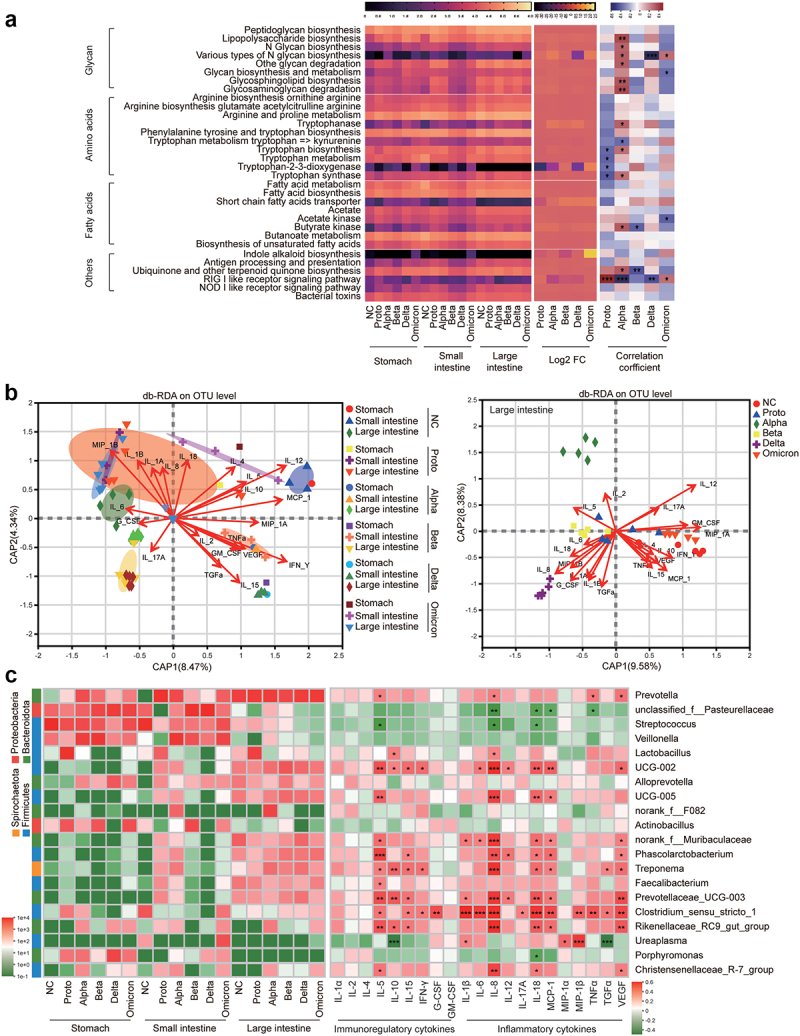
Prediction of microbial function and correlation analysis of tissue cytokines with microbiota in fragments of GI tract.

To investigate effects of GI microbiota on regulation of the immune microenvironment in the GI tract, we predicted metabolites and signaling pathways associated with infection and immunity on the basis of the microbiota in the GI tract fragments using the Phylogenetic Investigation of Communities by Reconstruction of Unobserved States (PICRUSt2), followed by analysis of their correlation with SARS-CoV-2 infection (Figure 4(a)). Overall, the predicted metabolites or associated pathways were primarily enriched in the large intestines of the GI tract, which positively correlated with of Alpha, Beta and Delta strains infections and negatively correlated with Proto and Omicron strains infections. Specifically, tryptophanase, a major immunoregulatory factor, was upregulated in the Alpha-, Beta- or Delta-infected animals and positively correlated with tryptophan biosynthesis. In contrast, fatty acid biosynthesis negatively correlated with Proto- or Omicron-induced infections. However, the SCFA transporter, acetate, and acetate kinase levels negatively correlated with most viral infections (except for Proto and Alpha). Additionally, Alpha, Beta and Delta strains negatively correlated with antiviral and inhibitory viral binding pathways, including the RIG-like receptor signaling pathway, which positively correlated with infections of Proto or Omicron strains (Figure 4(a)). These results suggested that distinct profile of GI microbiota caused by SARS-CoV-2 infections differentially produce metabolites that could be involved in regulation of the immune microenvironment in the GI tract.

(a) Correlations between predicted function of GI microbiota and infections of viral strains. Left panel, heatmap shows functions involved in immunity and infection of the KEGG pathway at level 3 predicted via the online tool PICRUSt2. Relative levels of abundance were expressed by color density in the scale bar above (log values of metabolite richness. The middle panel and heatmap show a log_2_ fold change (infected monkeys vs NC) of metabolite richness (mean). The relative levels of log_2_ fold change were expressed by color density in the scale bar above, which was plotted by the software GraphPad prism 8. The right panel and heatmap show correlation analysis of function profile of GI microbiota with infections of viral strains. Levels of correlation were expressed by color density in the scale bar above. Significant difference was marked with * (* *p* < .05, ** *p* < .01, *** *p* < .005, **** *p* < .001). The figure was plotted via R pheatmap package. Correlations were evaluated using the spearman correlation coefficient. (b) Correlation analysis of tissue cytokines with GI fragments via db-RDA analysis on basis of microbiota (OTUs) in fragments of GI tract, which was plotted via R vegan rda package. (c) Correlation analysis of tissue cytokines with GI microbiota. Left panel, heatmap shows abundance of the phylum (left ribbon) and top 20 microbial genera in fragments of GI tract, plotted via R vegan package. Right panel, heatmap shows correlation of the top 20 microbial genera with tissue cytokines. The significance was marked with *(* *p* < .05, ** *p* < .01, *** *p* < .005, **** *p* < .001), which was plotted via R pheatmap package. Correlations were evaluated using the spearman correlation coefficient.

Distance-based redundancy analysis (db-RDA) was performed to further explore the correlation among microbiota, tissue cytokines and viral infections in the GI tract fragments. Tissue cytokines distinctly correlated with infections caused by the corresponding viral strains in various GI tract fragments. In the large intestine, infection with the Alpha strain strongly correlated with IL-2, and Beta and Delta infections were highly associated with multiple inflammatory cytokines (IL-1β, IL-6, IL-8, IL-18, TGF-α and MIP-1α). In contrast, NC and Proto- or Omicron-infected primarily correlated with multiple immunoregulatory cytokines (IL-4, MIP-1α, TNF-α, GM-CSF, INF-γ) (Figure 4(b)). Furthermore, the top 20 most abundant genera were used to explore correlations between specific microbes and tissue cytokines in GI tract fragments (Figure 4(c)). Microbes that correlated with immunoregulation and inflammatory cytokines were primarily localized in the large intestine. Specifically, *Lactobacillus* was mainly correlated with IL-10 and IL-8 levels in the Proto-infected and NC animals. *Treponema* in the large intestine of ABD-infected monkeys significantly correlated with inflammatory cytokines (IL-8, IL-18, IL-1, TGF-α and VEGF) and immunoregulatory cytokines (IL-5, IL-15, and IL-10). *Clostridium sensu stricto 1*, an opportunistic pathogen, significantly correlated with nearly all tested cytokines, and showed a higher abundance in infected monkeys (except in Alpha-infected animals) than in NC animals. In conclusion, infections of SARS-CoV-2 Proto or its VOCs resulted in alternations of structure and composition of GI microbiota, which is indeed involved in immune responses to SARS-CoV-2 infection, possibly via their metabolites to modulate levels of inflammatory cytokines.

### Immune-related genes in GI tissues differentially activated post SARS-CoV-2 proto or its variants infection

**Figure 5.**
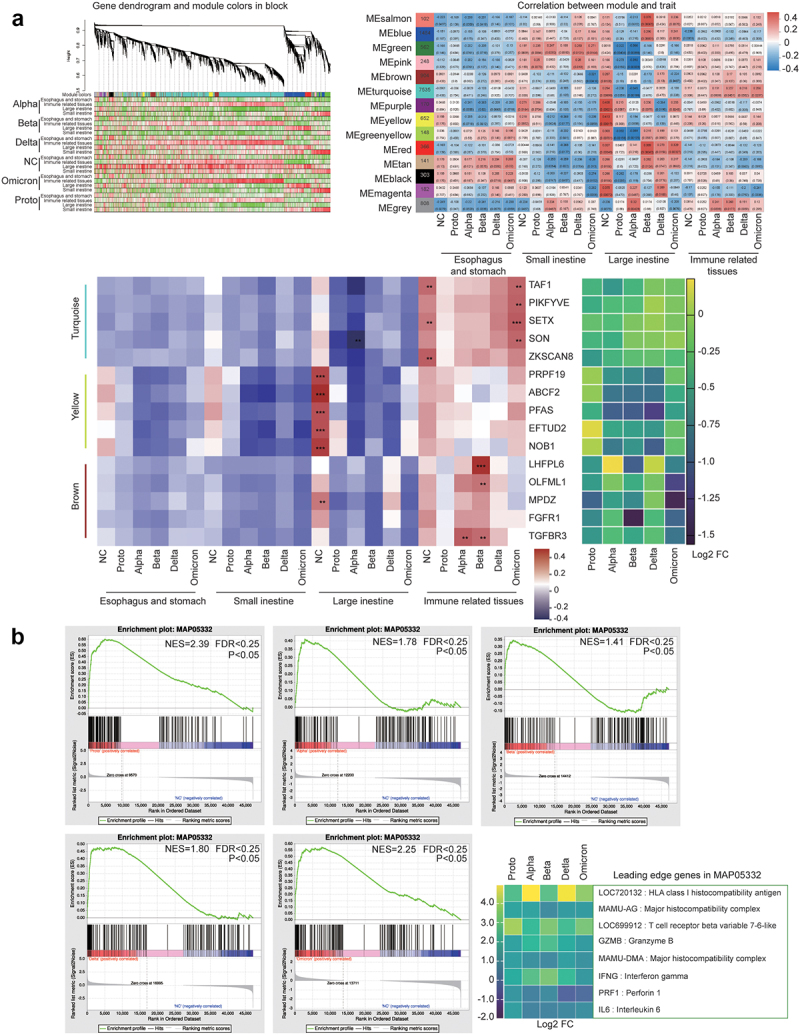
Transcriptomic profile of fragments in GI tract post SARS-CoV-2 or variants infection.

To further understand the GI response to SARS-CoV-2 infection, we performed a transcriptomic analysis of spleens and GI fragments from monkeys. After quality check 40,085 genes were identified, and the RNA-seq results were validated by qPCR (Fig. S7A). Other parameters for sequencing data base number and GC content) were presented in Supplementary Table S3. Compared with gene expressions in the NC animals 13,855 differentially expressed genes (DEGs) were identified in the spleens and GI tissues of SARS-CoV-2-infected animals. The KEGG analysis revealed that these DEGs were primarily enriched in pathways associated with signal transport, cancer, viral infectious diseases, and bacterial infectious diseases (Fig. S7B). Ranking of the DEGs analysis showed that *LOC706246* among the top 10 upregulated genes were upregulated in all infected animals (Fig. S8). Furthermore, *G6PD*, *NOX*1, and *LDHA*, associated with *LOC706246*, were upregulated in the GI tissues of all infected animals (Supplementary Table S2), suggesting that SARS-CoV-2 infection activated inflammatory genes in GI tissue via the glycolysis pathway. Distinct correlations were observed between DEGs and non-infection or infection of SARS-CoV-2 or its variants in the turquoise, yellow, and brown modules (Figure 5(a)). The top five hub-genes from each of the three modules showed strongly positive correlations primarily with immune-related tissues, including the Peyer’s patches, MLN, and spleen. In the Yellow module, autophagy- and immune-related genes (*PRPF9, ABCF2* and *EFTUD2*) showed lower expressions in ABD-infected animals than in PO-infected animals. In contrast, in the Brown module, five genes (*LHFPL6*, *OLFML1*, *MPDZ*, *FGFR1*, and *TGFBR3*) were more highly expressed in the ABD-infected animals than in PO-infected animals. Gene set enrichment analysis (GSEA) revealed that the DEGs were enriched in the infection- and immunity-associated pathways MAP05332. The critical genes in this pathway included *LOC720132* for HLA class I histocompatibility antigen, *MAMU-AG* for major histocompatibility complex, *LOC699912* for T-cell receptor beta invariable 7–6-like, *GZMB* for granzyme B, *IFNG* for interferon gamma, *PRF1* for perforin 1, and *IL6* for interleukin 6, the majority of which was upregulated in the GI tract of Alpha-, Beta- or Delta-infected animals (Figure 5(b)). Finally, matrix-correlation analysis was conducted on DEGs enriched in infectious pathways via rank analysis, WGCNA, and GESA according to their KEGG and GO functional annotations. DEGs were enriched in five clusters, including interleukin, integrin and addressin, antibacterial, antiviral, and inflammatory bowel disease(IBD)-related genes (Fig. S9). As mentioned above, these genes were primarily expressed in immune-related tissues (the spleen, Peyer’s patches, and MLN). Most of immunoregulatory cytokine genes were negatively correlated with non-infection in NC animal and positively correlated with Alpha, Beta or Delta infection (Fig. S9A). Integrin and addressin are important immune cofactors, most of which were positively correlated in GI tissues in infected animals but not in NC animal (Fig. S9B). Antibacterial and antiviral-related genes showed positive correlations in the immune-related tissues of VOCs-infected animals (Fig. S9C and D). In particular, some toll-like receptor genes showed significantly positive correlation in infected animals (Fig. S9D). Meanwhile, cytokine genes such as *IL2, IL10*, and *IL15*, associated with innate immunity against bacterial infection, mostly showed positive correlations in the GI immune-related tissues of infected animals. Notably, antiviral genes in the large intestines of Beta- or Delta-infected animals showed significantly positive correlations. Whereas, these genes showed significantly negative correlations in the Proto- or Omicron-infected animals. Additionally, genes associated with IBD showed significantly positive correlations in the immune-related tissues of VOC-infected animals. Meanwhile immune-related and antiviral genes in GI tissues (particularly large intestine and immune-related tissues) were transcriptionally activated, which may contribute to controlling viral infection or regulation of GI inflammation and pathogenic microbes (Fig. S9E). Results here showed that in GI tissues, differential expressions of immune-related genes were induced by infection of SARS-CoV-2 Proto or its variants. In particular, anti-viral genes were activated at the late stage of SARS-CoV-2 infection.

(a) Weighted gene co-expression network analysis (WGCNA) was performed to identify the top DEGs and their correlations with viral strains and fragments of the GI tract, following mRNA sequencing. Top panel, DEGs that were correlated with fragments of GI tract were clustered to form several modules as indicated (minModuleSize:30; minKEMtoStay:0.3; mergeCutHeight:0.25). Heatmap shows the module correlation of each gene (each row represents a GI fragment and each column represents a gene within the module). Correlation levels were differentiated by color (red means positive correlation and green means negative correlation). Middle panel, correlation of modules with specific phenotypes (GI segments). The numbers in the left column (modules) indicate the number of genes in the module. The numbers in the middle box indicate the correlation coefficient and *p*-value (in parentheses) between module and phenotype. Correlation levels were differentiated by color scale bar on the right (red for positive correlation and green for negative correlation). Bottom panel on the left, analysis of correlation matrix between the module genes (turquoise, yellow and brown) and GI tract. Levels of correlation were expressed by color density as shown in the scale bar. The significance was marked with * (*p < .0 5, **p < .01, *** *p* < .005, **** *p* < .001). Bottom panel on the right, heatmap of log_2_ fold change (infected monkeys vs NC) of values of module genes (mean). (b) Gene set enrichment analysis (GSEA) was conducted to identify leading edge genes as described in Materials and Methods. DEGs (NES >1.5 and *p* < .05) significantly enriched in MAP05332 (pathway associated with infection and immunity) were shown in plots, each of which represents an individual viral strain. Bottom panel on the right: heatmap shows log^2^ fold change of values (infected monkeys vs NC) of leading edge genes in MAP05332.

### Interactions between GI microbiota and host immunity involved in pathogenesis of SARS-CoV-2 proto or its variants infection

To further examine the potential effects of altered microbiota and transcriptional regulation of host genes on antiviral infection, we analyzed the correlations among GI-dominant microbiota, immune-related genes, and antiviral genes. In infected animals, the GI microbes with increased abundance (*Veillonella, Treponema* and *Clostridium sensu stricto 1*) showed strong positive correlations with interleukins (Fig. S10A), integrin and addressin, and antiviral genes (Fig. S10B). Furthermore, genes related to antibacterial activity and IBD were positively correlated with other GI microbes with increased abundance in infected animals, including *UCG-002*, *UCG-005*, and *Phascolarctobacterium* (Fig. S10C). In particular, GI tracts of Alpha-, Beta-, or Delta-infected animals showed a high abundance of *UCG-002, UCG-005*, *Phascolarctobacterium* and *Treponema* that were highly positively correlated with levels of interleukin genes (*IL16*, *IL17* and *IL15*) and integrin and addressin genes (*CCL13*, *CCL28*, and *CCR1*). *Rikenellaceae RC9 gut group* showed high abundance in Proto- (small intestine) or Omicron- (small and large intestines) infected animals, but not in Beta- or Delta-infected animals, which positively correlated with some interleukin genes (Fig. S10A). Notably, these microorganisms were abundant in the GI tracts of Alpha-, Beta-, or Delta-infected animals and showed highly positive correlations with antiviral genes, particularly *OAS1* that was upregulated in all infected animals. *Lactobacillus* abundance in the GI tracts of NC or Proto-infected animals showed no significant or negative correlation with antiviral genes and immunity-related genes. Additionally, the antiviral gene *LCN2*, which was upregulated in the GI tracts of Alpha-, Beta-or Omicron-infected animals, showed positive correlations with *Veillonella*, *UCG-002, UCG-005, Treponema* and *Clostridium sensu stricto 1* (Fig. S10B). *TLR9* and *DEFB* that are associated highly with antimicrobial functions, showed positive correlations with microorganisms of *F082*, *Phascolarctobacterium*, *Treponema*, and *Prevotellaceae UCG-003*. Among the IBD-associated genes, *RELA* showed a significant positive correlation with the genera *UCG-002*, *UCG-005*, *Phascolarctobacterium*, *Prevotellaceae UCG-003* and *Rikenellaceae RC9 gut group*. Thus, these results suggest that alterations in the GI microbiota and metabolites could be involved in the pathogenesis of SARS-CoV-2 infection via their interaction with host immunity.

## Discussion

Since the first report of SARS-CoV-2 infection, SARS-CoV-2 has been continuously evolving, which contributes to rapidly emerging variants, particularly VOCs (Alpha, Beta, Delta and Omicron) that possess distinct characteristics from the SARS-CoV-2 Proto strain.^26^ However, the immune cofactors and microbiota in the GI tract following infection with the SARS-CoV-2 prototype strain or its variants have not been comprehensively analyzed. In this study, we performed an integrated analysis of SARS-CoV-2 prototype strain or variant infections, GI microbiota, and host immune responses based on our previously established NHP model of SARS-CoV-2 infection,^7^ and revealed that all viral strains used in this study induced histopathological damage to the respiratory and digestive systems and altered the GI microbiota, and strains of SARS-CoV-2 in this study formed two distinct clusters, one with Alpha, Beta and Delta strains and the other with Proto and Omicron strains. To the best of our knowledge, this is the first study to systematically explore the GI environment in the NHP rhesus monkeys, demonstrating that the GI microbiota (distribution, abundance, and diversity) and mucin in each fragment of rhesus monkeys GI tract are similar to those in humans under the same physiological or pathological state.^22^ Furthermore, there are several advantages of NHP model study over human study. First, using the NHP model, we can test five viral strains of SARS-CoV-2 in monkeys with the basically consistent background at one time. However, it is impossible to find individual COVID-19 patient infected with five strains of viruses since only one or two strains cause one pandemic. Secondly, it is very hard to justify the infection stage during sampling in COVID-19 patients. Importantly, we can obtain content from each fragment of GI tract for microbiota analysis in the NHP model, which is hard to be performed in COVID-19 patients. We can only get feces samples from human patients. However, microbiota in feces is very different from those in other fragments of GI tract and cannot comprehensively reflect microbiota in GI tract. Therefore, the NHP is an ideal model for studying the GI manifestations of COVID-19 or other diseases.

The digestive tract consists of the esophagus, stomach, small intestine (duodenum, jejunum, and ileum), large intestine (cecum and colon), and rectum. Each fragment plays distinct roles owing to their different cellular composition and microenvironment. For example, the small and large intestines contain a large number of lymphoid tissues, which play regulatory roles under physiological or pathological conditions via interactions between microbiota or their metabolites and GI tissues.^24^ Dysbiosis of the GI microbiota is associated with multiple diseases and poor prognosis.^9^ Infections in extra-GI tissues can also cause dysbiosis of the microbiota in the GI system, which leads to complications in the GI tract or other systems.^27^ For convenience, feces were the most common samples used for analysis of the GI microbiome due to their similarity to that of microbiota in the rectum (Fig. S5E). However, as the distance from the rectum increases, the difference in the microbiota composition between the contents of GI fragments and feces also increases. Therefore, feces may not completely reflect the structure and diversity of microbiota in other fragments of the GI tract. For instance, *Faecalibacterium* and *Alloprevotella* were mainly abundant in the large intestine, and *Streptococcus* was mainly enriched in the small intestine, which is rarely detected in feces (Fig. S5E). Importantly, these three genera are involved in the pathogenesis of some diseases ,^28–30^ and could be potential biomarkers. Therefore, in this study, contents from each fragment of the GI tract collected for microbiome analysis, instead of feces only, should produce comprehensive profiles of microbiota in the GI tract.

Mucin, which is secreted by the goblet cells that are derived from intestinal stem cells at the bottom of the crypt, plays an important role in protecting GI tissues from microbial invasion.^31^ Mucin inhibits coronavirus infection via its glycan in an organoid *in vitro* model.^32,33^ Mucin mRNA in the blood or respiratory tissue could be a marker for SARS-CoV-2 infection and COVID-19 severity.^34^ Our previous study showed that the number of mucin-secreting cells decreases in some GI tract fragments at the early stage of SARS-CoV-2 infection, but gradually increases at the late stage of infection in the NHP model, indicating that mucin correlates with the recovery of the disease caused by SARS-CoV-2 infection.^7^ In this study, we observed that in most GI tract fragments at 5 dpi, showed an increase in the number of secreting-mucin goblet cells compared with those in the NC animal, particularly in Alpha, Beta or Delta infections (Figure 1(d)). An increase in goblet cells in the respiratory and digestive tracts is frequently reported in viral infections,^35^ which could be a type of host defense against viral infections. Moreover minor damage to epithelial cells induces the proliferation of intestinal stem cells, some of which further differentiate into goblet cells.^36^ Specifically, mucin MUC1 and MUC13 act as decoy molecules that are associated with the regulation of the expression of inflammatory cytokines such as IL-8 and TNF-α to regulate epithelial inflammation in response to inflammatory and infectious stimuli.^37^ For example, increased expression of *MUC1* and *MUC13* aggravates gastrointestinal inflammation.^38^ In this study, a significant positive correlation between mucin (specifically *MUC1* and *MUC13*) and SARS-CoV-2 infection was observed in the stomach and small intestine of infected animals (Fig. S3E), which indicates mucin is involved in inflammatory response in the GI tissues after the infection of SARS-CoV-2 (especially Alpha or Delta strain). Furthermore, this partially explains the high levels of inflammatory cytokines in the GI tissue of the Omicron-infected animals. Therefore, this study suggests damaged GI epithelial tissue of NHP begin to recover at 5 dpi after SARS-CoV-2 infection, which is evidenced by upregulation of GI tight junction and permeability-related genes *TJP1*, *TJP2*, and *EPCAM* (Supplementary Table S2).

Alterations in the structure and composition of the GI microbiota have been documented in several clinical studies and animal models of SARS-CoV-2 infection, including an increase in opportunistic pathogens (*Clostridium hathewayi*, *Actinomyces viscosus*, and *Bacteriodes nordii*) and a decrease in beneficial bacteria (*Faecalibacterium prausnitzii*, *Eubacterium rectale*, *Ruminococcus obeum*) ,^39 ,^ .^40,41^ Consistently, in this study, we also observed alterations of the microbiota in the contents of GI fragments post SARS-CoV-2 infection, particularly in the Alpha, Beta, and Delta strains (Figure 3(a) & Fig. S6). Moreover, this phenomenon frequently occurs in patients with COVID-19 and Post-acute COVID-19 syndrome (PACS) patients.^42,43^ Indeed, we observed an increases in the abundance of opportunistic pathogens (*Veillonella*, and *Treponema*, known as biomarkers for COVID-19^44,45^ and GI inflammation-associated bacteria (*Clostridium sensu stricto 1*)^46^ in the GI contents of SARS-CoV-2-infected animals, and a decreased abundance of probiotics (*Lactobacillus*) (Figure 4(c)). *Treponema*, a genus of Spirochaetota, interacts with the immune system in the GI mucus to induce inflammation,^47,48^ and is regarded as a potential biomarker for COVID-19 that can differentiate mild and severe status.^45,49^ In this study, the microbial genera with increased abundance in the GI tracts of Alpha-, Beta-, or Delta-infected animals (*Pasteurellaceae, Veillonella, Treponema, UCG-002, UCG-005* and *Phascolarctobacterium*) were primarily correlated with 2′-5′-oligoadenylate synthetase 1 gene (*OAS1*) and NF-kB subunit gene (*RELA*) that are associated with immunity, anti-virual, and IBD (Fig. S10). *OAS1*, which encodes an IFN-inducible antiviral protein, is vital in anti-viral (inhibition of viral replication) and inflammatory immune responses, which has been demonstrated in West Nile virus,^50^ hepatitis C virus,^51^ dengue virus,^52^ chikungunya virus,^53^ and measles virus^54^ infections. Additionally, genetic polymorphisms of *OAS1* are associated with the susceptibility of the Han population to SARS-CoV-2 infection.^55^ RelA is a member of the NF-κB complex that is involved in the expression of cytokines and host defense in macrophages, which is critical for the early inflammatory response and innate immunity in the lungs.^56^ Hyperactivation of RelA/NF-κB plays a major role in IBD pathogenesis.^57^ Therefore, the alteration of the microbiota as a result of SARS-CoV-2 infection, particularly by the Alpha, Beta, or Delta variants, may induce and exacerbate the systemic burden via reported or unknown mechanisms.

It has been reported that the lung-gut axis is involved in the pathogenesis of several respiratory viruses, such as influenza A virus and respiratory syncytial virus.^58^ Infection of the respiratory tissues may lead to systemic inflammation, which modulates the GI microbiota or inflammatory environment. And in turn, alternation of GI microbiota can affect respiratory disease via GI immunoregulatory functions of microbial metabolites. Therefore, we hypothesize that infection of SARS-CoV-2 results in the alteration of the GI microbiota and histopathology, which may in turn regulate respiratory infection via the lung-gut axis. In the follow-up study, we will test the hypothesis of the lung-gut axis first in a hamster model of SARS-CoV-2 infection as NHP are limited and expensive. SARS-CoV-2 infection induces the production of multiple inflammatory cytokines (IL-2, IL-7, IL-10, M-CSF, G-CSF, GM-CSF, MCP-1, and TNF-α). This may further lead to a cytokine storm, which is recognized as one of the crucial factors associated with increased tissue damage and disease severity.^59,60^ The percentages of CD4+ or CD8+ cells are considered to be important indicator for the severity and progression of SARS-CoV-2 infection.^61^ In this study, we observed that in addition to the increase in inflammatory cytokines in the serum of infected animals, the CD4+, CD8+ or CD68+ cell abundance also increased in some tissues, especially in ABD-infected monkeys. Furthermore, inflammatory cytokines such as IL-1α, IL-15, IFN-γ, MCP-1, VEGF, and TGF-α were induced in the GI tissues primarily by ABD and Omicron strains (Figure 2(a, b)). Disturbance of the GI microbiota by SARS-CoV-2 is associated with a cytokine storm, possibly owing to harmful metabolites produced by opportunistic pathogens and a decrease in beneficial metabolites produced by probiotics.^62^ For example, short-chain fatty acids (SCFAs), one of the most important probiotic metabolites, promote differentiation of naïve T cells into Th2 cells,^63^ regulate chemokines production and release by neutrophils and endothelial cells, and regulate adhesin expression.^64^ The abundance of GI microbes that produce SCFAs is usually negatively correlated with the biomarkers of systemic inflammation.^18^ *Lactobacillus* is an important microorganism that produces SCFAs (butyrate, propionate, and acetate).^65^ SCFAs-producing GI microbes that are affected by the upregulation of IL-15, induce inflammation in autoimmune and GI inflammatory diseases.^66,67^ Additionally, amino acids are important metabolites of the GI microbiota as they are associated with immunoregulation, including arginine, which is involved in inhibiting viral binding and replication.^68^ Tryptophan catabolism increases in the inflammatory microenvironment, promoting viral infection and survival.^69^ Tryptophan is also involved in the regulation of TGF-α and anti-inflammatory responses.^70^ In this study, SCFA-related functions negatively correlated with acetate, acetate kinase, and butyrate kinase functions, particularly in Beta-, Delta-, or Omicron infected animals (Figure 4(a)). Notably, IL-15 and TGF-α were upregulated in GI tracts of infected monkeys (Figure 2(a, b)). Importantly, the dominant microorganisms in the GI tract of the infected animals showed significantly positive correlations with IL-15 levels. Accordingly, we hypothesize that inflammation induced by SARS-CoV-2 infection disrupts intestinal homeostasis, and alters the structure and composition of the GI microbiota. Consequently, metabolites produced by the GI microbiota regulate immune responses that either cause inflammation or control viral infection. However, further metabolomic studies are necessary to clarify the mechanisms of microbiota alterations involved in pathogenesis of SARS-CoV-2.

Glucose 6-phosphate dehydrogenase (G6PD) is critical for the functioning of the antioxidant system and innate immune responses. G6PD produces NADPH via the pentose phosphate pathway, which further counteracts the oxidative stress induced by cytokine storms and inhibits inflammation.^71^ In this study, the ranking of DEGs in the GI tissues showed upregulation of the gene *LOC706246* (encoding fructose-bisphosphate aldolase) and its associated genes *G6PD, NOX1* and *LHDA* in GI tissues of all infected monkeys (Fig. S8 and Supplementary Table S2). Based on this, we propose that SARS-CoV-2 infection activates the anaerobic glycolysis process in the GI tract. G6PD is produced via the pentose phosphate pathway to upregulate NADPH expression, which maintains the oxidative stress response to control the cytokine storms in the GI tract. Additionally, carbohydrate metabolites produced by GI microbiota (particularly in the large intestine) of infected monkeys, such as glycans (glycosaminoglycan, N-glycan), may act synergistically with this process. N-glycan directly phosphorylates the D-mannose group of β-1,4-D-mannosyl-N-acetyl-D-glucosamine, which enters the glycolysis process.^72^ In this study, WGCNA showed that the DEGs of pro-inflammatory cytokines and antiviral immunity, including *SON*, *PRPF9*, *ABCF2*, and *EFTUD2* (Figure 5(a)), were downregulated in infected monkeys (Figure 5(a) and Supplementary Table S2). *SON* is involved in the regulating several type I IFN-inducible proteins and is associated with the inhibition of viral infection.^73,74^ *PRPF19*, *ABCF2* and *EFTUD2* also correlate with the genesis of some immune cells and production of cytokines ,^75 ,^ .^76,77^ Notably, genes associated with interleukins, addressin, and antivirus immunity are mainly correlated with GI immune tissues (Peyer’s patch, MLN) and spleen (Fig. S10A, B). Peyers patches and MLNs are important GI immune tissues that recognize pathogens and produce immune cells.^78^ However, correlation analysis showed that microorganisms highly correlated with antiviral and antibacterial immunity genes were mainly distributed in the large intestine, including *UCG-002*, *UCG-005*, *Treponema, Phascolarctobacterium* and *Clostridium sensu stricto 1* (Fig. S10B, C). These results indicate that antivirus immunity to SARS-CoV-2 infection primarily occurs in the small intestine (Peyer’s patches, MLN) and spleen. The large intestine regulates host immunity to SARS-CoV-2 infection mainly by adjusting the structure, composition, and metabolites of GI microorganisms.

Viral infection disrupts the integrity of the intestinal mucosa, which leads to damages to the GI barrier and allows the entry of pathogenic microbes into GI tissues. Additionally, LPS produced by harmful bacteria can enter the GI tissues and blood and induce the production of inflammatory cytokines.^62^ In fact, proteins of invasive microbes have been detected in the plasma of COVID-19 patients, and high levels of lipopolysaccharide-binding protein (LBP) have been observed in severe cases of COVID-19 patients.^79^ Toll-like receptors (TLRs) are a family of pattern recognition receptors that recognize pathogen-associated molecular patterns (PAMP), including bacterial LPS, which can induce the production of cytokines in infected epithelial cells and immune cells.^80,81^ In this study, we found that GI-dominant microorganisms correlated with genes involved in IBD and TLR, and the ratio of opportunistic pathogens increased (Fig. S10), indicating that infection with SARS-CoV-2 (particularly the Alpha, Beta, and Delta strains), evoke antimicrobial immune responses.

Rhesus monkey is well known as the best animal for R&D of vaccine and drug against SARS-CoV-2 and studies of viral pathogenesis. However, the rhesus monkey has become in short supply due to its overuse during the SARS-CoV-2 pandemic in the past few years. In addition, the high cost and strict ethics of rhesus monkey limit its application in basic research. Therefore, we can only get one monkey for each group, which is the shortcoming of this study. To compensate for this shortcoming, we meticulously designed this study and a comprehensive analysis was conducted to examine the impact of SARS-CoV-2 infection on gastrointestinal tissues and microorganisms. Some of results in this study are consistent with published data in human or animal studies. For example, the mucin levels gradually increased from the GI fragment duodenum to the rectum, which is consistent with data from the human GI tract. The abundance of opportunistic pathogens (*Veillonella, and Treponema*) increased in rhesus monkey after infection, which is known as biomarkers for COVID-19. Nevertheless, more animals, ideally hamsters, are required to validate the results of this study. Additionally, treatment with antibiotics and microbial metabolomics analysis are essential to clarify the pathogenesis of the GI microbiota in SARS-CoV-2 and variant infections. SARS-CoV-2 infection predominantly manifests as respiratory symptoms; hence, the mechanism of the gut – lung axis needs to be elucidated in the context of SARS-CoV-2 pathogenesis. In summary, this study has confirmed that rhesus monkeys are suitable NHP models for studying the regulatory mechanisms of the microbiota in human physiological or pathological conditions. Furthermore, the results of this study suggest that alterations in the GI microbiota induced by SARS-CoV-2 infection could be indirectly be involved in the pathogenesis of SARS-CoV-2 by producing infection- and immunity-associated metabolites. Notably, SARS-CoV-2 Proto and its variants in this study potentially cause distinct GI histopathology, inflammation, and microbiota (Table 1). These findings will be beneficial to precise prevention, control, and treatment of COVID-19.

**Table 1.** Summary of pathogenic characteristics in two clusters of SARS-CoV-2.

Characteristics	SARS-CoV-2 Clusters	
	PO	ABD
Severity of pathologic lesion		
*lung*	moderate	severe
*small intestine*	slight	moderate
*large intestine*	slight	severe
GI function-related		
Apoptosis (increased*)	slight	moderate
AB-PAS (increased*)	moderate	severe
ZO-1 (decreased*)	moderate^a^	moderate
EpCAM (decreased*)	slight	moderate
*viral load*	slight	severe
GI inflammation		
*Inflammatory cytokines*	slight^b^	severe
*Immunoregulatory cytokines*	slight	severe
CD4+ (increase*)	slight	Severe^c^
CD8+ (increase*)	slight	Severe^c^
CD68 (increase*)	slight	moderate
GI microbiota		
Probiotics (decrease*)	slight	severe
conditional pathogenic bacteria (increase*)	slight	moderate^3^
GI metabolites		
SCFAs (decrease*)	slight	moderate
Acetate (increase*)	slight	moderate
Tryptophanase (increase*)	null	severe
Arginine (increase*)	slight	moderate
Fatty acid biosynthesis (increase*)	slight	moderate
N glycan (increase*)	slight	moderate^d^
RIG-like receptor signaling pathway (increase*)	moderate	slight
NOD-like receptor signaling pathway (increase*)	slight	moderate

*Compared with NC animals.

^a^severely decrease in small intestine of Proto.

^b^multiple inflammatory cytokines were higher in Omicron.

^c^severely increase in Alpha and Delta.

^d^severely decrease in Delta.

## Ethics approval

All animal procedures in this study were approved by the Institutional Animal Care and Use Committee of the Institute of Medical Biology, Chinese Academy of Medical Science (ethics number: DWSP202101 001). All experiments involving live SARS-CoV-2 were conducted at the ABSL-3 laboratory of the National Kunming High-level Biosafety Primate Research Center.

## Consent for publication

All authors have consented to publication.

## Availability of data and material

All data generated or analyzed during this study are included in this published article and its supplementary information files.

The datasets generated and/or analyzed during the current study are available in the NCBI repository, including transcriptome sequencing data (accession number: PRJNA899127, https://submit.ncbi.nlm.nih.gov/subs/sra/SUB12236568/overview) and 16S rRNA sequencing data (accession number: PRJNA894807, https://submit.ncbi.nlm.nih.gov/subs/sra/SUB12210866/overview).

## Supplementary Material

Supplementary_materials_gut microbes_revise3.docx

Sup_table_3.xlsx

Sup_table 1 clean.docx

Sup_table_2.xlsx

Sup_table 1.docx

## List of abbreviations

ABD: Alpha, Beta and Delta
PO: Proto and Omicron
SARS-CoV-2: severe acute respiratory syndrome coronavirus 2
COVID-19: Coronavirus Disease 2019
MERS-CoV: Middle East respiratory syndrome coronavirus
VOCs: variants of concern
ACE2: angiotensin converting enzyme 2
GI: Gastrointestinal
MAIT: mucosal-associated invariant T
SCFAs: Short-chain fatty acids
DAMPs: danger-associated molecular patterns
RLRs: RIG-I-like receptors
NLRs: NOD-like receptors
NHP: non-human primate
ABLS3: Animal Biosafety Level 3
AB-PAS: Alcian blue and Periodic acid-Schiff
OTUs: operational taxonomic units
H&E: hematoxylin-eosin staining
PCoA: Principal coordinate analysis
PBS: phosphate-buffered saline
PLS-DA: partial least squares discriminant analysis
PFU: plaque- forming units
db-RDA: distance-based redundancy analysis
PICRUSt2: Phylogenetic Investigation of Communities by Reconstruction of Unobserved States
FPKM: fragments per kilobase of exon per million mapped reads
FDR: false discovery rates
DEGs: differentially expressed genes
GO: Gene Ontology
KEGG: Kyoto Encyclopedia of Genes and Genomes
WGCNA: weighted gene co-expression network analysis
GSEA: Gene set enrichment analysis
G-CSF: granulocyte colony-stimulating factor
GM-CSF: granulocyte-macrophage colony-stimulating factor
IFNγ: interferon gamma
TGF: transforming growth factor
TNF: tumor necrosis factor
VEGF: vascular epithelial growth factor
MCP: Monocyte Chemoattractant Protein
MIP: macrophage inflammatory protein
MLN: mesenteric lymph node
IBD: Inflammatory Bowel Disease
