# Supplementary material for: Gastrointestinal microbiota and metabolites possibly contribute to distinct pathogenicity of SARS-CoV-2 proto or its variants in rhesus monkeys
Source: Gut Microbes. 2024 Apr 2;16(1):2334970. doi: 10.1080/19490976.2024.2334970 (PMC10989708; doi:10.1080/19490976.2024.2334970)
Supplement: Supplementary_materials_gut microbes_revise3.docx [file KGMI_A_2334970_SM5066.docx]

**
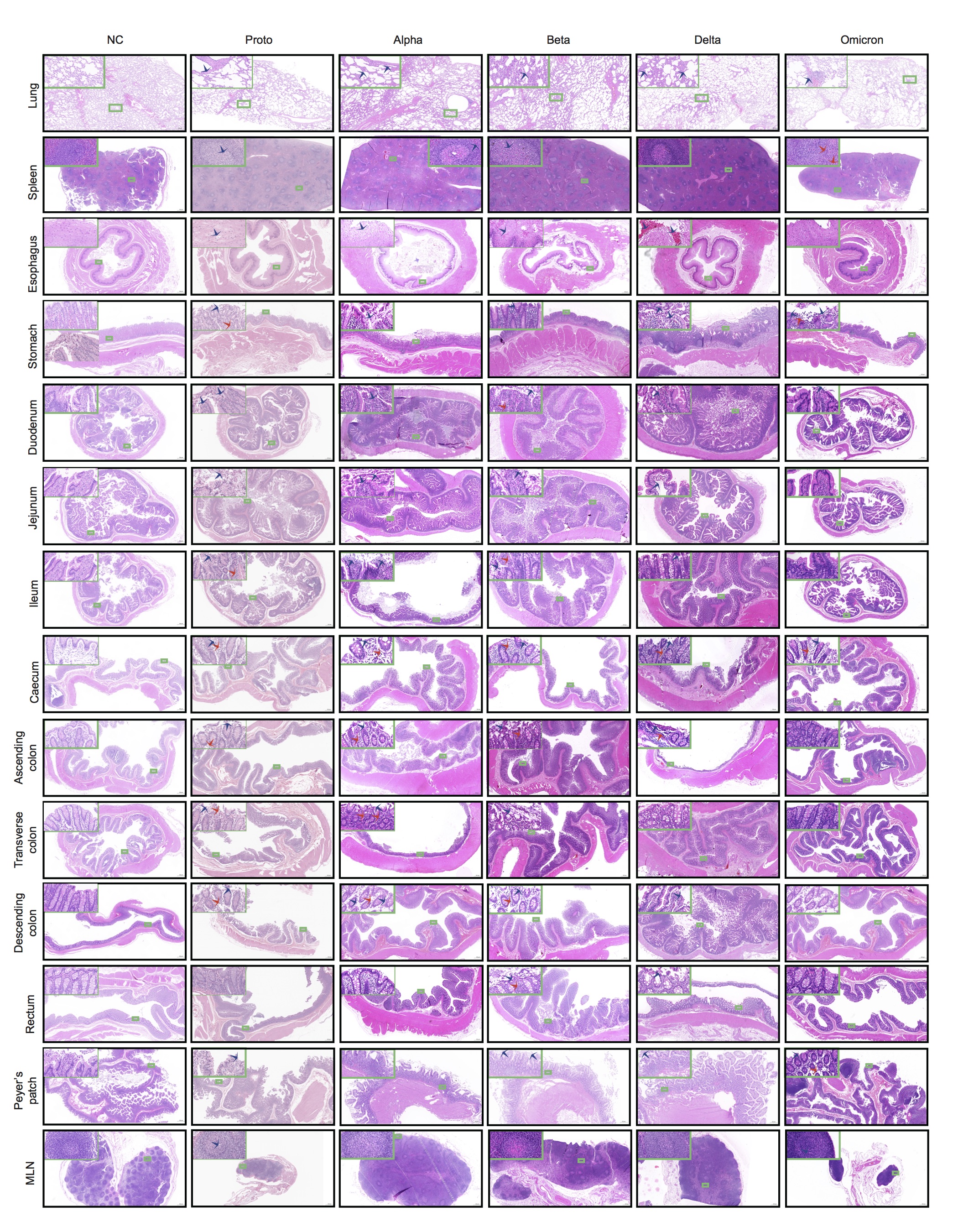
 Figure S1**

**Related to Fig. 1.** Histopathological analysis of pulmonary and GI tissues in rhesus monkeys challenged with SARS-CoV-2 or variants. On 5 dpi, animals were euthanized and dissected. The indicated tissues were harvested and processed for H&E staining and histopathological evaluation as described in Materials and Methods. The scale bars are 20μm.

**Figure S2**

(*A*) Viral genomic RNA was detected in tissues and faces of monkeys infected with SARS-CoV-2 prototype strain or variants. On 5 dpi, animals were euthanized and dissected. Tissues and GI contents/feces were collected for analysis of viral RNA via qRT-PCR as described in Materials and Methods. Heat map of gene expression levels of (*B*) *ACE2*, (*C*) *TMPRSS2* and (*D*) *ADAM7* in GI segment after infection with Proto and VOCs strains.

**
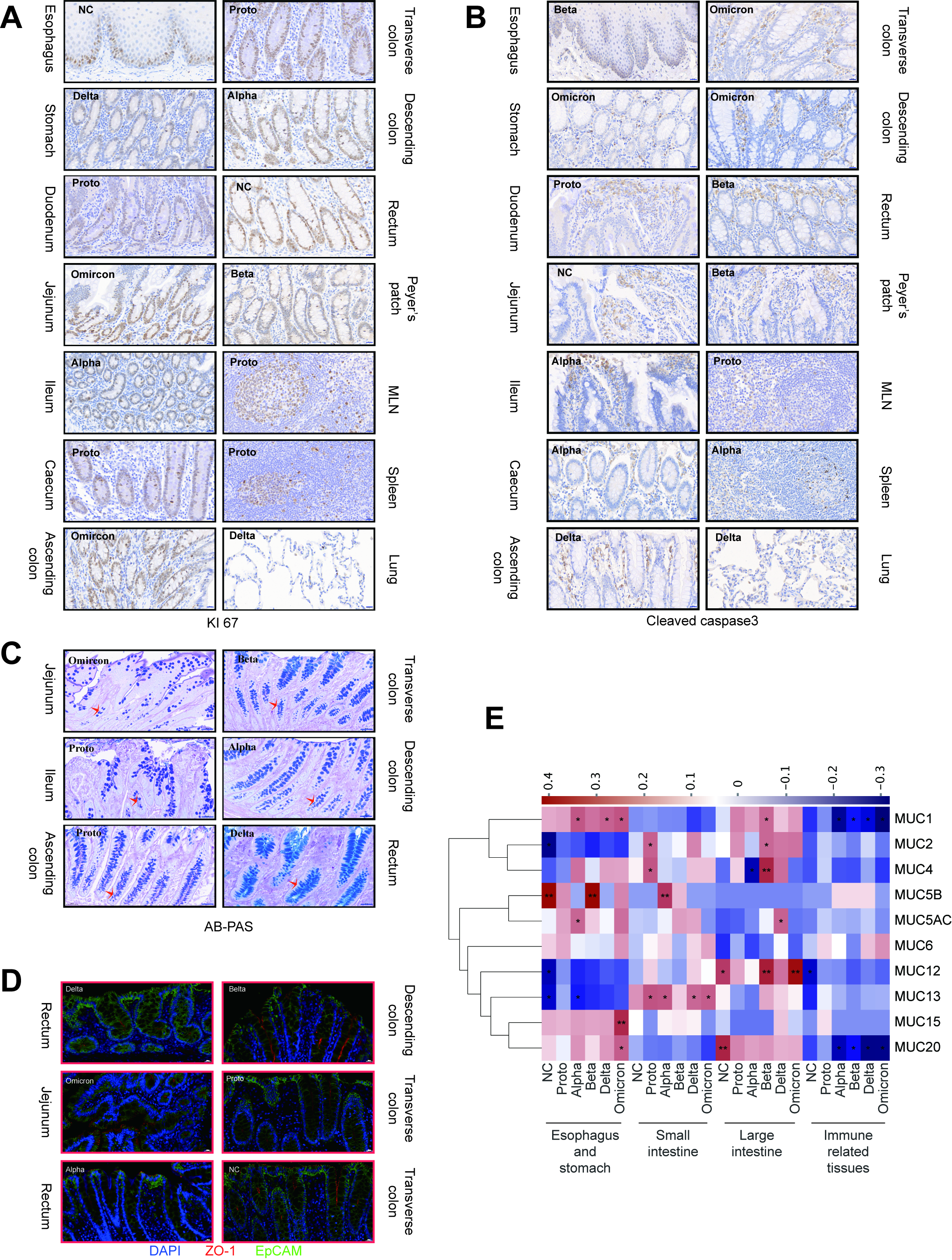
**

**Figure S3**

**Related to Fig. 1 and Fig. 2.** On 5 dpi, animals were euthanized and dissected. Tissues were collected for the following analyses. Representative images were shown here for IHC staining of Ki67 (*A*), cleaved caspase 3 (*B*), AB-PAS staining of mucin (*C*), immunofluorescence staining of Zonula occludens protein (ZO-1, red) and epithelial cell adhesion molecule (EpCAM, green) (*D*). The scale bars are 20μm. (*E*) Correlation analysis was conducted between key mucin protein genes in fragments of GI tract and SARS-CoV-2 or variants infection. Levels of correlation (*Spearman*) were expressed by color density in the scale bar above. Significance was marked with * (∗ P <0 .05, ∗∗ P < 0.01).

**Figure S4**

**Related to Fig. 2. A** Representative images for immunofluorescence staining of CD4+ (green) and CD8+ (red) cells. **B** Representative images for IHC staining of CD68. The scale bars are 20μm. Staining density was calculated as described in Methods.

**Figure S5**

**Related to Fig 3.** Microbial Alpha-diversity, Beta-diversity, composition and dominant microorganisms in GI contents and feces of NC monkey. (*A*) Sobs index of GI microorganisms in each intestinal segment. (*B*) Shannon index of GI microorganisms in each intestinal segment. (*C*) Heatmap shows abundance of the top 20 genera in the indicated fragments of GI tract, which was plotted via R vegan package. (*D*) Partial least squares discriminant analysis (PLS-DA) of GI microbiota. (*E*) Composition of microbes in the indicated fragments of GI tract at the genus level.

**Figure S6**

**Related to Fig 3.** On 5 dpi, animals were euthanized and dissected. GI contents were collected for 16s rRNA sequencing and analysis of microbiota. (*A*) Alpha-diversity index (Sobs and Shannon) of GI microorganisms in fragments of GI tract. The statistical differences between NC and SARS-CoV-2 infection were analyzed (*Kruskal-Wallis H test*; *P<0.05; **P<0.01; ***P<0.005; ****<0.001). (*B*) Relative abundance of potentially pathogenic microorganisms in GI contents of NC and infected rhesus monkeys.

**Figure S7**

**Related to Fig. S10.** (*A*) Quantitative real-time PCR (qRT-PCR) validation of differentially expressed genes (DEGs) by linear regression analysis, the linear regression value was: R^2^ = 0.955; (*B*) On 5 dpi, animals were euthanized and dissected. Tissues of GI tract were collected for RNA sequencing and transcriptomic analysis. DEGs were annotated in KEGG pathways between NC and SARS-CoV-2 infections. Each panel represents a comparison between NC and the corresponding strain of SARS-CoV-2 as indicated.

**
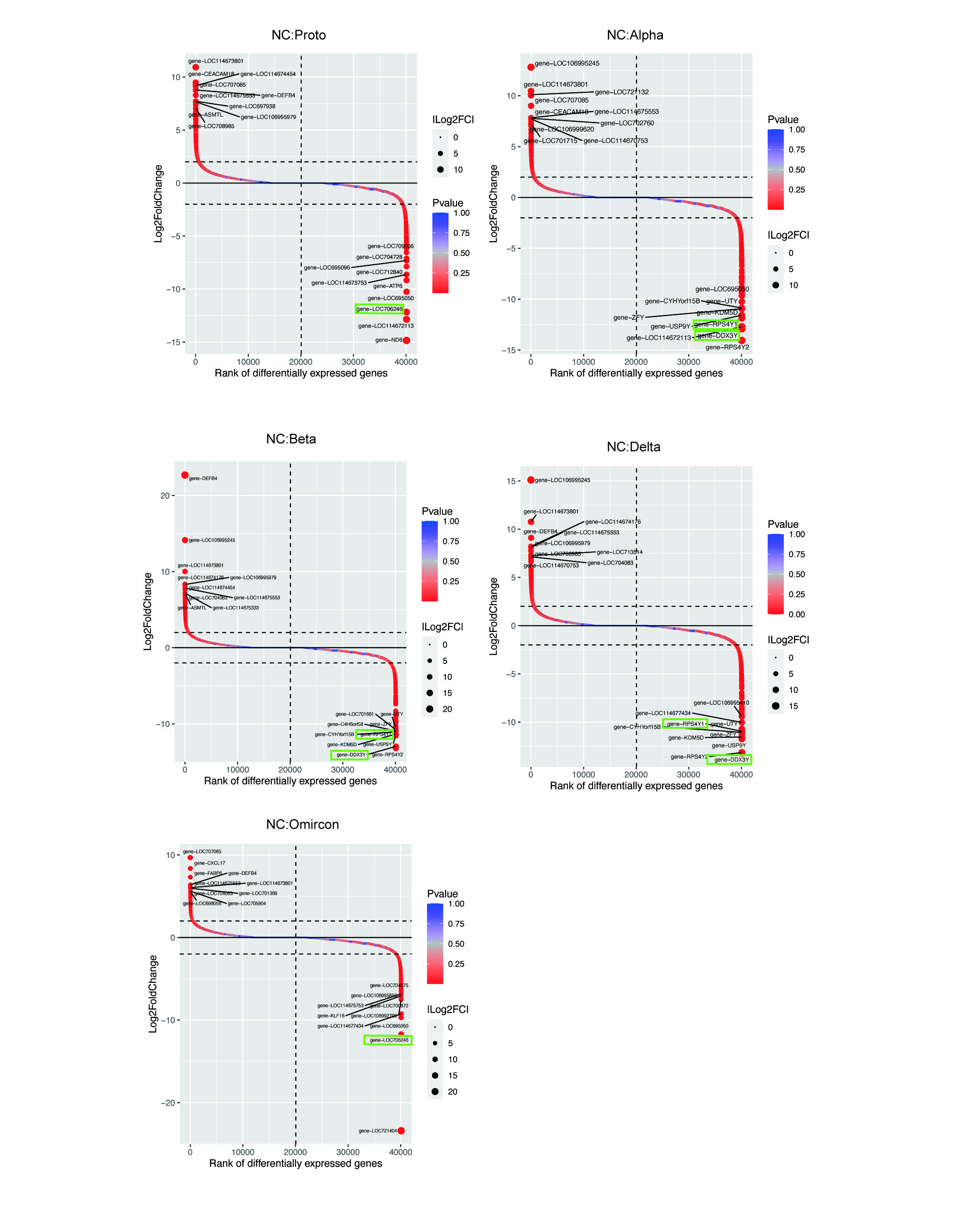
**

**Figure S8**

**Related to Fig. S10.** On 5 dpi, animals were euthanized and dissected. Tissues of GI tract were collected for RNA sequencing and transcriptomic analysis. Rank analysis of DEGs was performed between NC and strains of SARS-CoV-2 infection. Each panel represents a comparison between NC and the corresponding strain of SARS-CoV-2 as indicated.

**Figure S9**

**Related to Fig. S10.** On 5 dpi, animals were euthanized and dissected. Tissues of GI tract were collected for RNA sequencing and transcriptomic analysis. Correlation analysis was conducted between DEGs (infection and immunity-associated) in fragments of GI tract and SARS-CoV-2 or variants infection. (*A*) interleukin genes; (*B*) integrin and addressin genes; (*C*) antiviral genes; (*D*) antibacterial genes; (*E*) inflammatory bowel disease (IBD)-associated. Levels of correlation were expressed by color density as shown in the scale bar.


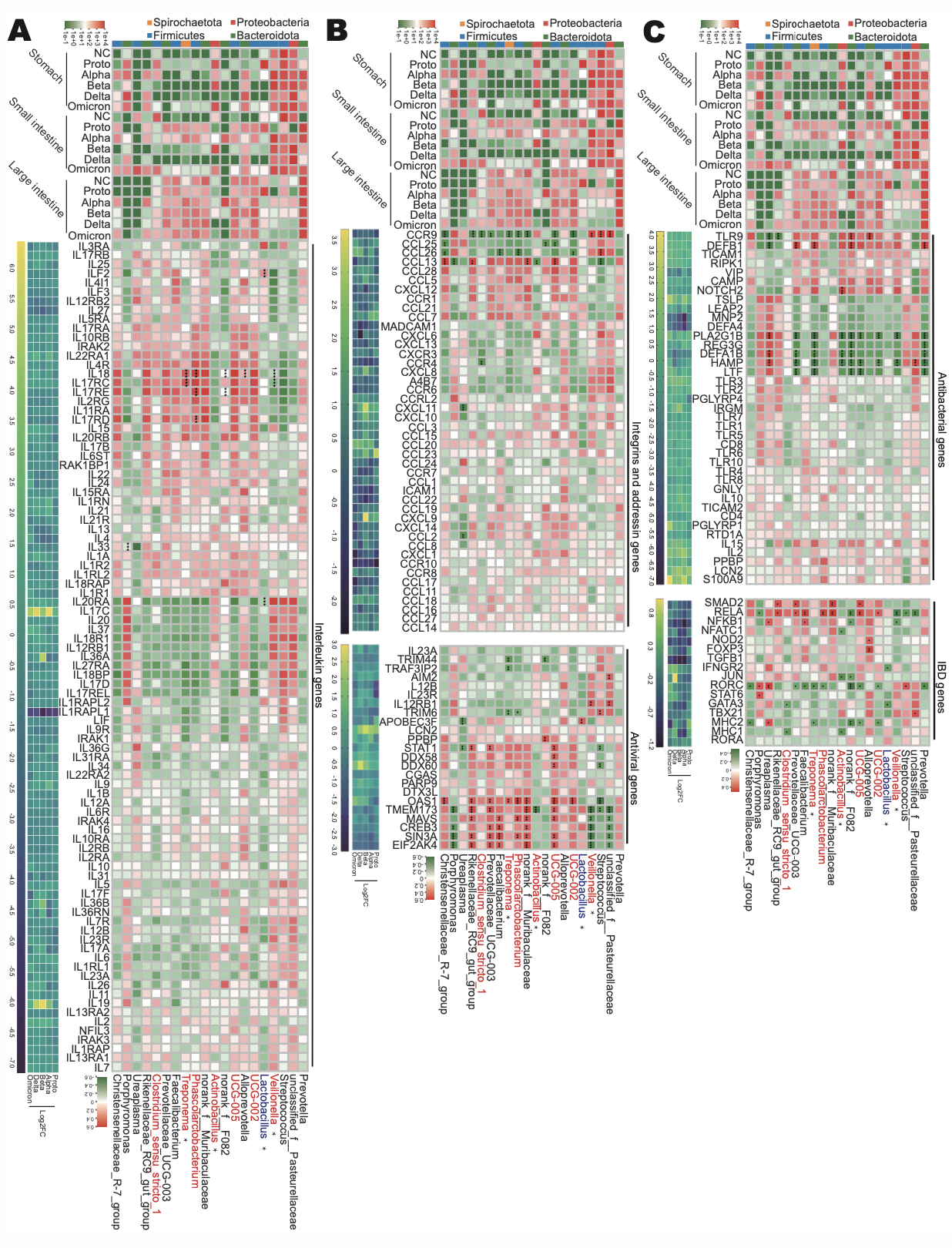
**Figure S10**

**Correlation of gene regulation levels and GI microbiota post SARS-CoV-2 infection.** On 5 dpi, animals were euthanized and dissected. Tissues were collected for transcriptomic analysis as described in Methods, followed by further correlation analysis of GI microorganisms with DEGs (**A** interleukin genes; **B** integrin and addressing, and antiviral genes; **C** antibacterial and inflammatory bowel disease genes, which was obtained via analyses of WGCNA, KEGG, GSEA and Rank analysis.) post SARS-CoV-2 infection. Each panel (**A,** **B** and **C)** consists of two parts. Heatmap on the left of each panel shows abundances of the four major phyla (left ribbon) and the top 20 genera (listed on the left of each panel where * (*p*<0.05) means significantly different between infected monkeys and NC), plotted via R vegan package. Heatmap (red-green) on the upper right of each panel shows correlation of differential microbial genera (Top 20) with indicated DEGs. Levels of correlation were expressed by color density as shown in the scale bar. The significance was marked with * (∗ P < 0 .05, ∗∗ P < 0.01, ∗∗∗ P < 0.005, ∗∗∗∗ P < 0.001), plotted via R pheatmap package. Correlations were evaluated using the spearman correlation coefficient. Heatmap (blue-yellow) on the bottom right of each panel shows log_2_ fold change (infected monkeys vs NC) of values of differentially expressed levels (mean).
