# Supplementary material for: Gastrointestinal microbiota and metabolites possibly contribute to distinct pathogenicity of SARS-CoV-2 proto or its variants in rhesus monkeys
Source: Gut Microbes. 2024 Apr 2;16(1):2334970. doi: 10.1080/19490976.2024.2334970 (PMC10989708; doi:10.1080/19490976.2024.2334970)
Supplement: Sup_table 1 clean.docx [file KGMI_A_2334970_SM5064.docx]

Supplementary Table 1

**Animal information and change of clinical information after infection**

| **Animal NO.** | RM1 | RM2 | RM3 | RM4 | RM5 | RM6 |
| --- | --- | --- | --- | --- | --- | --- |
| **Gender** | Male | Male | Male | Male | Male | Female |
| **Age** | 5 | 13 | 10 | 7 | 10 | 6 |
| **Virus** | NC | Proto | Alpha | Beta | Delta | Omicron |
| **CT value** |  | | | | | |
| Day 0 | 0 | 0 | 0 | 0 | 0 | 0 |
| Day 1 | 0 | 18.16 | 22.79 | 17.35 | 20.98 | 17.22 |
| Day 3 | 0 | 23.81 | 15.53 | 21.76 | 19.80 | 18.75 |
| Day 5 | 0 | 23.78 | 13.76 | 22.91 | 15.72 | 18.48 |
| **BWC(g)** | Body weight change(n dpi vs 0 dpi ) | | | | | |
| Day 0 | 0 | 0 | 0 | 0 | 0 | 0 |
| Day 1 | +27 | -38 | -30 | -145 | -410 | -370 |
| Day 3 | +4 | -6 | +10 | -30 | +160 | -640 |
| Day 5 | +138 | -50 | -300 | -300 | -200 | -1040 |
| **BTC** | Body temperature change (n dpi vs 0 dpi) | | | | | |
| Day 0 | 0 | 0 | 0 | 0 | 0 | 0 |
| Day 1 | +0.1 | +1.3 | +1 | +1.7 | +0.5 | +0.6 |
| Day 3 | +0.1 | -0.2 | +1.3 | +0.9 | 0 | -0.1 |
| Day 5 | 0 | -0.3 | -0.2 | +0.5 | -0.5 | -0.6 |
